# Protecting Companion Animals Under Chinese Criminal Law: Current Practice and Future Paths

**DOI:** 10.3390/ani16142119

**Published:** 2026-07-08

**Authors:** Chunyan Liu, Da Su, Anzi Nie

**Affiliations:** 1 School of Law, Henan University of Science and Technology, Luoyang 471023, China; chunyan@haust.edu.cn; 2 Postdoctoral Research Station in Law, School of Law, Xiamen University, Xiamen 361005, China; sdepl@xmu.edu.cn; 3 School of International Law, Zhongnan University of Economics and Law, Wuhan 430073, China

**Keywords:** companion animals, animal cruelty, criminal regulation, indirect legal protection

## Abstract

China’s legal system currently lacks a comprehensive animal protection law, and its Criminal Law contains no specific offense for animal cruelty. Consequently, judicial protection for companion animals relies on offenses designed to safeguard human interests, such as intentional destruction of property, theft, and releasing hazardous substances, manifesting a distinct characteristic of indirect protection. This study systematically reviews criminal cases to examine the current state of animal protection provided by Chinese criminal law, identifying the fundamental limitation that such protection is merely incidental or reflective. The research findings indicate that while current frameworks are human-centric, there is significant untapped potential within existing statutes. Specifically, this article advocates for utilizing the crime of illegal use of information networks and other relevant offenses to address the critical regulatory gap concerning the production, sale, and dissemination of animal torture media. This pragmatic solution offers a valuable reference for jurisdictions facing similar legislative voids where specialized animal protection laws are unlikely to be enacted in the short term, promoting a more effective utilization of existing criminal doctrines to safeguard animal welfare.

## Simple Summary

The current Criminal Law of China lacks a specific offense for animal cruelty, which means the protection of companion animals is only achieved through indirect legal paths. Currently, judicial authorities can only protect animals incidentally by applying charges designed to safeguard human interests, such as “intentional destruction of property,” “theft,” or “releasing hazardous substances.” Meanwhile, a rampant underground industry involved in the production, sale, and dissemination of animal torture media remains in a regulatory vacuum. This article argues that, given the immediate challenges of establishing direct animal protection statutes, it is crucial to tap into the potential of existing criminal charges. Specifically, this article explores the potential of utilizing existing criminal charges—such as the crime of illegal use of information networks and other relevant offenses—to regulate the dissemination of animal torture media, thereby addressing a critical deficiency in the current criminal justice system regarding the protection of companion animals.

## 1. Introduction

At present, China lacks a comprehensive animal protection law. Instead, provisions on animal welfare remain scattered across various laws and regulations. Since 2009, the Institute of Law at the Chinese Academy of Social Sciences (CASS) has drafted the “Animal Protection Law of the People’s Republic of China (Experts’ Draft Proposals).” As many critics argued that this draft was overly broad in scope and lacked practical operability, the Institute subsequently drafted the “Anti-Animal Cruelty Law of the People’s Republic of China (Experts’ Draft Proposals)” in 2010 [1]. Despite persistent and unremitting appeals from various sectors of society through multiple channels, neither the “Animal Protection Law (Experts’ Draft Proposals)” nor the “Anti-Animal Cruelty Law (Experts’ Draft Proposals)” has officially entered the legislative agenda of China’s national legislature to date.

On the one hand, at the national legislative level, the Wildlife Protection Law is the only statute dedicated to the subject; however, its scope is strictly limited to wildlife, excluding companion animals [2]. Other relevant laws either reflect animal protection values only minimally or lack them entirely. On the other hand, at the level of administrative regulations, animal protection is often only indirectly reflected through specific measures. While the implementation regulations for terrestrial and aquatic wildlife briefly address rescue efforts in principle [3,4], the Regulations on the Administration of Laboratory Animals are a rare exception. Article 27 explicitly mandates that staff must care for laboratory animals and strictly prohibits mistreatment or abuse [5]. Nonetheless, this remains limited to the narrow context of laboratory settings.

Overall, animal protection provisions within the current Chinese legal framework are sparse and largely indirect, with significant regulatory gaps persisting in the domain of companion animal welfare. At present, only certain local “Dog Management Regulations” explicitly prohibit the abuse and abandonment of companion animals. For instance, both the Beijing Dog Management Regulations and the Shanghai Dog Management Regulations introduce a principled prohibition against the abuse or abandonment of dogs, yet they fail to establish corresponding legal consequences [6,7]. Similarly, while the Nanjing Dog Management Regulations, Suzhou Dog Management Regulations, and Liaoning Province Dog Management Regulations do prescribe punitive measures for such acts, these remedies are limited to minor administrative fines [8,9,10]. However, due to a lack of robust enforcement mechanisms or the leniency of existing penalties, these regulations function as “toothless tigers.”

The confluence of extensive legislative voids and negligible legal costs has effectively neutralized any deterrence against the abuse or even mutilation of companion animals in China. Nevertheless, such acts have increasingly surfaced in the public eye through the media in recent years, with particularly egregious incidents igniting fierce public outcry and ethical debates across society.

The landmark case of “Beijing’s First Criminal Prosecution for Pet Poisoning” serves as a pivotal example. In September 2022, the defendant, retaliating against neighborhood dogs for urinating on and scratching his electric scooter, scattered poisoned bait within a residential compound. The act resulted in the poisoning of eleven pet dogs and two stray cats (of which nine dogs and two cats died). While initially handled by the police as “intentional destruction of property,” the case was eventually investigated and prosecuted under the charge of “releasing hazardous substances.”

In December 2025, the Beijing Chaoyang District People’s Court delivered the first-instance judgment, sentencing the defendant to four years in prison. The court explicitly noted that conflicts arising from “uncivilized dog ownership” do not justify extreme acts of poisoning or animal cruelty, emphasizing that social harmony encompasses the relationships between humans, animals, and the environment.

However, the civil compensation aspect reveals a persistent judicial logic: pets are legally categorized as mere property. One victim, who suffered from clinical depression following the loss of their dog, sought 250,000 RMB in emotional distress damages, arguing that companion animals—as sentient beings—cannot be equated with ordinary objects. Nevertheless, forensic appraisal institutions refused to value the pets, citing a “lack of assessment parameters.” The court’s initial ruling awarded only partial material damages while dismissing all claims for emotional distress. After the procuratorate declined a protest (appeal for retrial), five plaintiffs appealed the civil portion of the judgment. On 16 April 2026, the appellate court upheld the original ruling, once again rejecting the claims for emotional damages [11,12,13,14].

This case pointedly reflects the “indirect” logic inherent in the protection of companion animals under Chinese criminal law. Although the defendant was punished, the conviction—“the crime of releasing hazardous substances”—falls under the category of “crimes against public safety.” Its core objective is to safeguard the personal safety of indeterminate persons, rather than the lives of animals. As noted by Rajbangshi, modern criminal justice systems are largely underpinned by “anthropocentric assumptions” that prioritize harms to humans while relegating animal cruelty to the periphery of legal concern [15]. In other words, the law protected companion animals only tangentially, by shielding the people. The root of this indirectness lies in the absence of dedicated statutes criminalizing companion animal abuse or mutilation. Nevertheless, the case demonstrates that existing criminal law can be mobilized to protect companion animals even without specialized provisions.

Inspired by this case, this article suggests that while a national law for companion animal protection remains a necessary long-term goal, the current legislative void should not lead to inaction. Instead of waiting for new statutes, it is more practical to explore how the existing Chinese Criminal Law can be utilized to its fullest extent. When a specialized legal solution is not yet available, the priority should be to make the best use of the criminal law tools already at hand. Human society should adopt a high moral ground by treating all animals with equality and humanity [16]. This stance is rooted not only in our intuitive and scientific recognition of animal sentience and their capacity for suffering, but also in the extension of human moral concern to non-human species [17]. Empirically, moral obligation serves as the most potent predictor of an individual’s intention to intervene in instances of animal abuse [18]. This does not imply that animals possess a moral status equivalent to that of humans; rather, it acknowledges that they hold distinct moral significance. Consequently, legal policymakers and practitioners are duty-bound to incorporate animal interests into their decision-making processes [19].

The primary research aim of this paper is to systematically examine the current landscape of companion animal protection within the Chinese criminal justice system and, while respecting the established doctrinal boundaries of the existing criminal law, to explore the latent potential of underutilized or “dormant” statutory provisions that could be mobilized to protect companion animals in the future. To achieve this objective, this study seeks to address two core research questions. First, it investigates the operational status and structural limitations of companion animal protection under the current Chinese criminal law. Second, it explores what potential or adaptable provisions exist within the current criminal law framework that can be legally interpreted to regulate emerging forms of animal-related misconduct.

The structure of this paper is organized as follows: Section 3 uses case studies to show how China’s criminal justice system currently protects companion animals under existing laws. Section 4 connects social reality with legal theory to explore “dormant” provisions in the Criminal Law—articles that have not yet been used for companion animal protection but have the potential to be applied. Beyond improving the rule of law for companion animal welfare in China, this paper aims to provide a practical and valuable framework for other regions facing similar legal gaps. It offers a strategic path forward for jurisdictions where passing specific animal welfare legislation remains difficult.

It should be pointed out that the concept of “companion animal” remains relatively unfamiliar within both the current legal system and the civil society of China. In existing Chinese legislative practice and official regulatory contexts, there is a distinct lack of precedents utilizing “companion animal” as a formal statutory term; similarly, in the daily discourse of the general public, the term has yet to achieve widespread recognition or common usage.

To clarify the conceptual boundary of this study, the definition of “companion animal” employed herein primarily draws upon both the expert draft proposals of Chinese academia and recent local legislative explorations.

First, this study refers to the definition of “pet animals” outlined in the Animal Protection Law of the People’s Republic of China (Expert Draft Proposal) and the Anti-Animal Cruelty Law of the People’s Republic of China (Expert Draft Proposal). These two draft proposals explicitly state that “pet animals,” also referred to as “companion animals,” encompass any domesticated animals owned by individuals or organizations for the purpose of personal entertainment or companionship, including but not limited to cats, dogs, birds, and horses. Under these frameworks, once any animal not prohibited by law is kept as a pet, it is legally deemed a companion animal [1].

Second, closely following the latest local legislative dynamics, this study incorporates the Administrative Measures for the Protection and Management of Urban Companion Animals in Sanming City (Draft for Public Comment), released by the Sanming Municipal Justice Bureau on 27 March 2026. This document defines companion animals as dogs and cats routinely kept within domestic residences for non-economic purposes such as companionship and psychological comfort [20].

In conclusion, synthesizing Chinese academic theoretical achievements and recent local regulatory practices, this study defines a “companion animal” as any domesticated animal kept by individuals or organizations for non-economic purposes, such as psychological comfort and companionship, provided that such possession is not prohibited by law.

## 2. Materials and Methods

The research materials for this study were gathered from four primary sources. First, we conducted a comprehensive search of China’s current legal framework via the National Database of Laws and Regulations and the National Library of Government Regulations. This allowed for a systematic review of animal protection provisions—specifically regarding companion animals—across various levels, including national laws, administrative and local regulations, and judicial interpretations.

Second, criminal cases were retrieved from the Wolters Kluwer database, which includes official judgments from courts across all 31 provinces in mainland China. On 24 May 2026, a systematic search for criminal cases was conducted in the database. The search used the following settings: the full-text search keywords were “宠物” (pet) OR “狗” (dog) OR “犬” (dog, the formal word for “dog” in Chinese) OR “猫” (cat) OR “鸟” (bird) OR “鹦鹉” (parrot) OR “猪” (pig) OR “鱼” (fish) OR “龟” (turtle) OR “仓鼠” (hamster) OR “兔” (rabbit) OR “鸭” (duck) OR “鹅” (goose) OR “鸽” (pigeon) OR “蛇” (snake) (Note: These terms cover the most common pets and companion animals in Chinese judicial practice. Since the types of pets are endless in reality, any rare species not listed here are highly likely to be caught by the first word “宠物” [pet], which courts commonly use in their descriptions); the judgment date was limited from 24 May 2019 to 23 May 2026; and the case type, document type, and trial procedure were limited to “criminal,” “judgment,” and “first instance.”

This search found 2706 judgments, including 2697 standard criminal judgments and 9 judgments with attached civil actions. After reviewing these documents, we identified 16 different charges under the Chinese Criminal Law where the cases involved harm to companion animals. These charges are listed in Table 1 by their article numbers in the Chinese Criminal Law. To ensure accessibility for an international readership, the relevant cases were translated and summarized into English. During this process, the translation of all specialized legal terms and specific charges strictly adhered to the official English version of the Criminal Law published by the Legal Affairs Commission of the National People’s Congress (NPC) of China.

It should be noted that the following discussion will not cover the offenses of “Smuggling rare animals” (Table 1, No. 8) and “Crimes of endangering precious or endangered wild animals” (Table 1, No. 16). The former protects precious animals prohibited from import and export by the state, while the latter safeguards wildlife under special state protection; neither bears a genuine legal connection to the protection of companion animals.

Additionally, we collected public news reports from major online platforms to track detailed information and public sentiment regarding animal cruelty incidents, with a particular focus on the underground industry involved in producing and distributing animal abuse media. Finally, academic monographs and journals provided the theoretical foundation for our analysis.

Regarding methodology, this study adopts a mixed-methods research design that integrates quantitative empirical case analysis with qualitative legal doctrinal interpretation. Quantitatively, we systematically categorized the retrieved criminal cases into statistical groups (such as those presented in Table 1). For each case, we recorded basic legal facts, including the specific criminal charges applied, the nature of the offenses, and the final sentencing outcomes. This statistical sorting allows us to present a macro overview of the current judicial trends regarding how companion animals are treated under the existing criminal law. Qualitatively, we use standard doctrinal legal analysis to examine specific cases, focusing on how courts interpret the elements of crimes and explain their judgments. Furthermore, this paper will analyze specific articles within the existing Criminal Law that have the potential to be applied to companion animal protection in the future.

Because making a brand-new, specialized animal protection law in China faces long-term difficulties, the goal of our study is to find practical solutions within what we already have. By carefully analyzing the specific elements of existing crimes, this paper explores how the current Criminal Law can be used to protect companion animals right now, without waiting for major legislative changes.

## 3. Current Status of Companion Animal Protection in China’s Criminal Justice System

**Figure 1:**
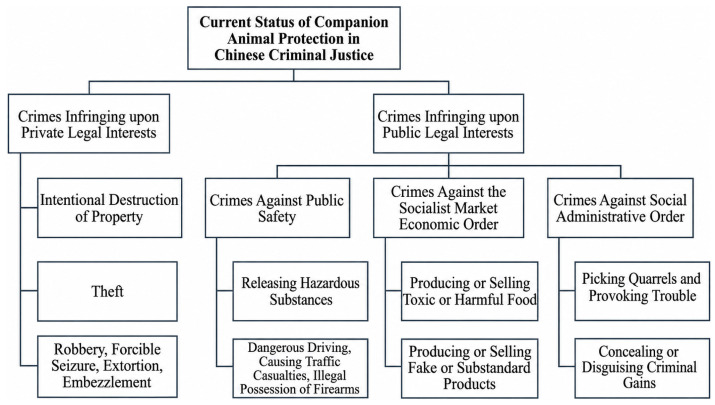
Current Status of Companion Animal Protection in Chinese Criminal Justice.

In criminal law theory, a crime is defined as an act that infringes upon legal interests, which are fundamentally divided into private and public interests. Private interests pertain to individual welfare, such as life, physical integrity, liberty, and property. In contrast, public interests encompass collective welfare, including national security, public safety, and economic or social order. Accordingly, criminal offenses are classified based on whether they infringe upon private or public legal interests. As illustrated in Figure 1, the following sections provide a systematic analysis of how these two categories of offenses function to protect companion animals within the Chinese criminal justice system.

To clarify the theoretical foundation of the framework illustrated above, it is necessary to theoretically define the concepts of “indirect,” “incidental,” and “reflective” protection used throughout this study. Although these three terms overlap in describing non-direct animal protection, they represent distinct legal dimensions under the current criminal law framework.

Indirect protection primarily focuses on the specific legal path. Because companion animals are not recognized as independent legal subjects in current Chinese statutes, the existing legal system can only shield them indirectly by safeguarding the human owner’s property rights or maintaining public order.

On the other hand, incidental protection and reflective protection describe the outcomes and jurisprudential nature of this framework. Incidental protection focuses on the practical outcome of judicial enforcement. When a court punishes animal cruelty under property or public safety crimes, the primary goal of the judgment remains the protection of human interests, while the physical safety of the animal is achieved merely as an accidental byproduct. Meanwhile, reflective protection captures the jurisprudential essence of these laws, meaning that while the statutory rules are designed purely to protect humans, their strict enforcement objectively projects a reflective benefit that covers the existence and welfare of companion animals.

### 3.1. Protection of Companion Animals via Crimes Infringing upon Private Legal Interests

#### 3.1.1. Protection of Companion Animals via the Crime of Intentional Destruction of Property

The crime of intentional destruction of property is codified under Chapter V, “Crimes Against Property,” of the Specific Provisions of the Chinese Criminal Law [21]. As illustrated in Table 2, nine cases were identified in which companion dogs were the primary or partial targets of intentional destruction. In these instances, the fundamental basis for conviction is the legal recognition under Chinese criminal law that the intentional killing of another person’s animal constitutes the intentional destruction of property [22].

Analysis of these nine cases reveals two primary deficiencies in the protection of companion animals through the charge of intentional destruction of property.

First, the valuation of companion animals is frequently marginalized compared to other physical assets. Specifically, in Cases 1, 2, and 3, where the criminal acts targeted both companion dogs and other property, the courts failed to specify the independent value of the dogs involved. This article argues that since the pet dogs involved are regarded as the property of the victims, their value should be determined as clearly as that of any other property involved in the case.

This ambiguity is particularly problematic given that the Chinese Criminal Law is characterized by a dual-threshold system that defines offenses based on both qualitative nature and quantitative scale. In certain scenarios, the specific value of a companion dog may be the decisive factor in determining whether the total damage meets the “relatively large amount” threshold required for criminal prosecution [23]. Even when other damaged assets already meet this threshold, the valuation of the companion dog remains indispensable: it dictates the overall social harm of the act and directly influences the sentencing outcome. In contrast to Cases 1–3, the valuations were explicitly conducted in Cases 4–9, where the destructive acts were directed solely at the animals. This disparity demonstrates that while methodologies or conclusions may be debated, the valuation of companion dogs in judicial practice is entirely feasible and should not be circumvented.

Secondly, while current judicial practices protect companion animals as personal property, they fail to signal to the public that poisoning dogs for theft—regardless of the motive—triggers multiple criminal charges. A clear distinction exists within the identified cases. While Cases 1–4 are straightforward instances of intentional destruction of property, Cases 5–9 involve an additional element of theft. In these latter instances, the defendants followed a “poison-then-steal” pattern, driven either by personal consumption (Cases 5, 8) or illicit profit (Cases 6, 7). The victims of such trafficking eventually end up either on dining tables or in the hands of animal abusers. Despite this dual criminality, only the judgment in Case 7 explicitly recognized the concurrence of both intentional destruction and theft.

Regarding the legal characterization of this “poison-then-steal” pattern, three doctrinal boundaries must be clarified under criminal law theory before establishing its nature. First, it does not constitute an apparent concurrence. The intentional destruction of property and theft protect entirely distinct legal interests; there is no relationship of specialty or overlap between their statutory provisions, leaving independent conceptual space for each crime to be convicted. Second, this conduct cannot be covered by the traditional doctrine of absorption. While some referenced judgments only convicted the defendants of a single offense by treating the poisoning as a mere incidental result absorbed into the theft, the prior act of poisoning stands as an independent method behavior and cannot be logically absorbed by the subsequent theft. Third, it does not equal an ideal concurrence of crimes. An ideal concurrence requires the execution of a single physical act. In this pattern, however, delivering the lethal bait and subsequently returning to carry the carcass away are factually and temporally separate actions, constituting multiple behaviors rather than a single act.

Consequently, this pattern is best evaluated under the framework of implicated offenses. In Chinese criminal law doctrine, an implicated offense arises when a defendant executes a crime whose method or result behavior concurrently violates another statutory provision. Instead of applying concurrent punishments, courts are required to choose the single heavier offense for conviction and sentencing [24]. Crucially, an implicated offense can only be established when a specific means is typically employed to commit a specific end-crime, or when a causal behavior routinely leads to a particular consequential behavior [22]. In practice, specialized dog-stealing heavily relies on the “poison-first” modality; therefore, such conduct inherently touches upon both the intentional destruction of property and theft, satisfying the dogmatic criteria of an implicated offense.

Furthermore, this implicated relationship must be systematically recognized regardless of whether the subsequent theft is successfully completed or remains an attempt. Among the sampled pool, a stark inconsistency emerged. In Case 8, the court applied the implicated offense framework exclusively to instances where the defendants failed to carry the dogs away after poisoning them, whereas successful asportations were labeled solely as theft. Conversely, in Cases 5, 6, 8, and 9, because the defendants failed to carry the poisoned dogs away, the courts restricted their legal evaluations entirely to the intentional destruction of property. We argue that this bifurcation is doctrinally flawed. The failure to secure the physical property is invariably driven by external factors beyond the defendant’s control, such as being interrupted by residents. Such fortuitous contingencies should not dictate or alter the legal nature of the underlying criminal conduct.

This stance finds implicit regulatory alignment in the judicial interpretation of theft issued by the Supreme People’s Court. The interpretation mandates that “employing destructive means to steal public or private property, thereby causing destruction to other property, shall be penalized severely as theft; where the conduct concurrently constitutes both theft and another crime, courts shall apply the heavier offense” [25]. Structurally, this provision targets scenarios where a perpetrator destroys separate properties to facilitate theft, such as smashing a car window to steal valuables inside. Under Chinese criminal law, the intentional destruction of property requires a minimum statutory threshold of financial damage. If the car window’s value falls short of this threshold, the first half of the provision applies (severe punishment under theft); if it meets the threshold, the second half triggers (choosing the heavier offense). While the “poison-then-steal” pattern targets the stolen companion animal itself rather than auxiliary property, this divergence remains merely superficial. In both scenarios, the underlying criminal logic is identical: the perpetrator deploys destructive means to realize an illicit possessory end. Therefore, when destructive means deployed during theft simultaneously satisfy the statutory elements of property destruction and theft, the doctrine of implicated offenses must be applied.

This systematic omission of “multiple offense” recognition undermines the conduct-guiding function of criminal law and fails to deter the rampant “poison-and-steal” black market. Theoretically, criminal law serves not only to protect legal interests but also to regulate and shape public behavior through judicial rulings. These rulings act as a mirror through which the public understands state prohibitions. If courts were to explicitly state that poisoning dogs for theft constitutes both property destruction and theft, it would foster a robust social deterrent. Such a stance would convey that comprehensive criminal liability is inevitable, regardless of the motive or whether the poisoning and theft were “successful.” Ultimately, a more rigorous and comprehensive judicial intervention is necessary to disrupt these illicit supply chains.

#### 3.1.2. Protection of Companion Animals via the Crime of Theft

The crime of theft is codified under Chapter V, “Crimes Against Property,” of the Specific Provisions of the Chinese Criminal Law [21]. This study identified 65 cases of dog theft occurring between 2022 and 2025, with representative instances summarized in Table 3. Similar to the cases discussed previously, many of these incidents involve the “poison-then-steal” pattern, raising the same issue of concurrent offenses.

Interestingly, Cases 5–9 occurred earlier (between 2014 and 2019). The data suggests a shifting trend in Chinese judicial practice: when dealing with dogs stolen via poisoning, courts now increasingly favor a conviction of theft over intentional destruction of property. However, this “either-or” approach remains incomplete. As argued above, a comprehensive legal evaluation should recognize the concurrence of both theft and property destruction.

Furthermore, the determination of “theft while carrying a deadly weapon” has emerged as a critical issue, particularly within the rampant black market for dog trafficking. In cases where defendants use crossbows to fire poisoned darts or anesthetic needles, judicial consensus is lacking. Some courts categorize such conduct as “theft while carrying a deadly weapon” (e.g., Cases 10 and 11), while others do not (e.g., Cases 12 and 13).

Under Chinese Criminal Law, the crime of theft comprises five primary modalities: (1) theft of property involving a “relatively large amount”; (2) multiple thefts; (3) burglary (theft by entering a residence); (4) theft while carrying a deadly weapon; and (5) pocket-picking. Notably, the latter four categories are not subject to the “relatively large amount” monetary threshold for criminal prosecution.

According to judicial interpretations issued by the Supreme People’s Court and the Supreme People’s Procuratorate, “theft while carrying a deadly weapon” refers to the possession of instruments prohibited by the state—such as firearms, explosives, or controlled knives—or any other instrument capable of endangering personal safety during the commission of a crime [25]. Crucially, the interpretation establishes an “either/or” relationship between these two categories. While the latter requires a case-by-case assessment of concrete danger based on the instrument’s purpose and nature, the former—state-prohibited instruments—carries a legislative presumption of severe risk.

Prevailing legal theory in China holds that this designation does not require the perpetrator to display, imply, or actually use the weapon against a victim [22]. It is sufficient that the instrument is inherently dangerous or capable of assault. Even if the perpetrator possesses the weapon solely for use on objects or has no intention of using it at all, the criterion is met as long as the possession is intentional [26].

In Chinese judicial practice, the 2011 Amendment (VIII) to the Criminal Law introduced “theft while carrying a deadly weapon” primarily because such conduct inherently threatens personal safety, even if the perpetrator’s immediate target is property [27].

To address concerns regarding the over-extension of this criminal category, tools should be classified based on their nature and risk profile: (1) state-prohibited instruments (e.g., firearms, regulated knives), which carry an inherent legislative presumption of lethal risk due to their strict regulation by the state; (2) instruments used exclusively against animals (e.g., standard bird nets or traditional iron traps), which pose no immediate threat to human life and must be excluded from the “deadly weapon” category; and (3) instruments that, while practically deployed against animals, possess immediate collateral lethality capable of endangering humans.

In cases where defendants use crossbows firing poisoned or anesthetic darts to steal companion animals, the act satisfies the criteria for “carrying a deadly weapon” under this third category. First, under Chinese law, a crossbow is not a standard tool but a strictly regulated instrument; its unauthorized possession violates the Public Security Administration Punishment Law [28].

Opposing scholars argue that if a perpetrator uses a crossbow solely to incapacitate animals without the subjective intent to confront humans or resist arrest, it should not be deemed a deadly weapon [29]. However, this study contends that such a view overlooks the objective situational danger of these instruments. A “deadly weapon” is defined by its capacity to engender an intense apprehension of physical peril in the public consciousness [22], carrying an abstract risk that does not require an explicit intent to assault humans [30].

More importantly, combining high-power crossbows with darts laced with highly toxic chemicals amplifies the potential threat to human safety. Although few judgments explicitly detail the chemical composition, real-world data and exceptional cases confirm the rampant use of cyanide, sodium cyanide, and succinylcholine. Under China’s Hazardous Chemicals Catalogue and the Hazardous Chemicals Safety Law (2025), individuals are strictly prohibited from purchasing these acute toxins. A high-velocity crossbow loaded with deadly poisons is completely distinct from traditional, low-risk hunting tools used exclusively against animals. Should a confrontation arise with pet owners, local residents, or law enforcement officers, these toxic projectiles can be instantly redirected, presenting a fatal threat to human life.

In judicial practice, confirming whether such specialized tools constitute “deadly weapons” heavily relies on establishing objective evidence regarding the precise cause of animal death and the exact chemical mechanisms used. To this end, the rapid development of veterinary forensic pathology provides technical and evidentiary support for judicial authorities. Pathological examinations and forensic necropsies can scientifically interpret tissue lesions to verify the severity and nature of the violence inflicted on companion animals [31]. Furthermore, advanced histological evaluations and ancillary techniques—such as the sodium rhodizonate test used to detect metallic residues from projectiles—allow investigators to precisely identify gunshot or chemical dart entry wounds, thereby transforming covert black-market operations into undeniable forensic proof in court [32].

In summary, the determination of “theft while carrying a deadly weapon” should center on whether the tool’s objective dangerousness induces a profound sense of physical insecurity in an ordinary person. For perpetrators who deploy highly toxic crossbows and heavy metal tongs as their “industry standard,” elevating their charges to aggravated theft ensures robust deterrence against the “poison-steal-sell-consume” black market, even when individual cases fail to meet monetary thresholds.

#### 3.1.3. Protection of Companion Animals via Other Crimes Against Private Interests

Beyond the crimes of intentional destruction of property and theft, the Chinese criminal justice system also provides protection for companion animals through other offenses targeting private interests, such as robbery, forcible seizure, extortion, and embezzlement. However, compared to the primary charges of property destruction and theft, these offenses play a marginal role in animal protection. This is due not only to the scarcity of relevant cases identified in this study but also to the fact that companion animals often occupy a peripheral position in the factual circumstances of such crimes. Table 4 summarizes the representative cases identified in this study regarding these additional categories of offenses.

Furthermore, another relevant charge is the crime of concealing or disguising criminal gains [21]. Although this is technically a “fencing” crime directed at public interests, its close proximity to the aforementioned property crimes warrants its inclusion in this analysis.

In Case 14, although the defendant’s original intent was theft, the nature of the offense escalated to robbery after they employed coercive threats against the victim upon being discovered [21]. In Case 15, the act of forcible seizure was primarily driven by the exploitation of the victims’ vulnerability as minors. Regarding Case 16, the extortion involving a pet dog was merely one link in a broader chain of criminal activities; notably, the court consolidated multiple offenses to identify the defendants as an “evil forces” crime syndicate, meaning that the targeting of companion animals served as just one manifestation of the syndicate’s organized illicit operations. Finally, as for Case 17, a more precise interpretation is that Chinese criminal justice “potentially” provides protection for companion animals through the crime of embezzlement. This distinction is crucial because the court ultimately declined to convict the defendant, ruling the case inadmissible due to insufficient evidence and the existence of a civil fee dispute.

Cases 18 and 19 demonstrate that the black market for dog theft and illicit trade is sustained by a mutual “consensus” between buyers and thieves. Consequently, legal interventions—whether targeting downstream fencing crimes or upstream property offenses—contribute to the protection of companion animals. The critical distinction between concealing criminal gains and joint theft hinges on the existence of “prior conspiracy”.

In Case 18, Defendant D was only convicted of concealing criminal gains because his involvement occurred ex post facto (after the theft was completed). In contrast, Defendants A and B in Case 19, despite not physically committing the theft, were identified as accomplices in joint theft due to their prior conspiracy with C. In practice, the stable cooperative ties within these criminal chains often imply a tacit prior agreement. Classifying professional buyers as accomplices in theft rather than mere fences significantly enhances the deterrent effect, as the Crime of Theft carries heavier statutory penalties and has a lower threshold for criminal prosecution in Chinese criminal law.

### 3.2. Protection of Companion Animals via Crimes Infringing upon Public Legal Interests

#### 3.2.1. Protection of Companion Animals via Crimes Against Public Security

The aforementioned “Beijing’s first criminal prosecution for pet poisoning” is widely regarded as a landmark case in the protection of companion animals within the Chinese criminal justice system. In fact, prosecutions for poisoning companion animals had occurred well before this case. This study identified nine such cases through a comprehensive search. In eight instances, the defendants directly targeted companion dogs; in the remaining case, the defendant originally intended to poison a human victim, but the victim’s vigilance led to the accidental death of the pet dog instead. Among the eight cases involving direct targeting, the defense in five instances challenged the legal classification of the offense as “the crime of releasing hazardous substances, the details of which are summarized in Table 5.

In the five cases discussed above, a significant controversy emerged regarding whether the defendants’ actions truly endangered public security. This debate stems from the fact that the defendants specifically targeted companion animals, and their actions resulted exclusively in animal fatalities without causing human casualties. This friction highlights the inherent challenges faced by the Chinese judiciary when utilizing “human-centric” offenses to protect companion animals.

According to Chinese criminal law doctrine, the legal interest protected under Chapter II (Crimes Against Public Security) of Criminal Law is “public security,” defined strictly as the life, safety, and physical health of an unspecified or majority of people. On one hand, “majority” is the core characteristic of the victims, while “unspecified” implies a realistic possibility that the harm could expand to a larger group at any time. On the other hand, if a conduct results only in property damage without endangering human life or health, it is generally not classified as a threat to public security [33].

In short, all offenses against public security, including the crime of releasing hazardous substances, are fundamentally characterized as crimes against “human safety.” In these cases, the defense argued that humans were unlikely to encounter the hazardous substances, rendering the conduct insufficient to threaten human safety. Conversely, the prosecution and the courts maintained that the administration of hazardous substances in public spaces created a sufficient risk to human safety to warrant criminal conviction.

This study contends that both “Beijing’s first criminal prosecution for pet poisoning” and the eight cases of releasing hazardous substances specifically targeting companion dogs share a critical commonality: the defendants placed hazardous substances in areas frequently accessed by residents, such as community lawns, elevators, staircases, sky gardens, and parking lot entrances. While the immediate consequences involved the deaths of companion or stray animals—appearing, at first glance, unrelated to human life or health—the potential for human harm remains significant.

Specifically, individuals interacting with their companion animals or feeding stray animals run a high risk of accidental exposure to these toxins. Furthermore, the risk of accidental contact is particularly acute for persons with limited or no capacity for civil conduct (e.g., children or the elderly), who may lack the judgment to identify such hazards. Therefore, the act of releasing hazardous substances in these environments inherently possesses the nature of endangering public security and fulfills the legal elements of the crime of releasing hazardous substances.

In summary, while the defense consistently argued that the poisoning was restricted to animals and resulted merely in property damage, the courts sustained convictions for the crime of releasing hazardous substances by drawing a clear boundary: whether the conduct objectively threatens the life, health, or property safety of an unspecified or majority of people. Although these offenses targeted companion animals, the boundary was crossed because perpetrators deployed strictly regulated, highly lethal chemical toxins within heavily trafficked public spaces. Under criminal dogmatics, evaluating this public hazard relies on the objective attributes of the act rather than the perpetrator’s subjective target restriction. Because these lethal substances were scattered in shared spaces, they generated an immediate, non-excludable physical threat to an indeterminate public, establishing a concrete risk rather than a merely hypothetical one.

Beyond the crime of releasing hazardous substances, the inclination of the Chinese judiciary to protect companion animals is also evident in judicial instruments involving other offenses against public security. These include drunk driving-related dangerous driving, traffic casualties, and the illegal possession of firearms [21], as detailed in Table 6. However, consistent with the cases involving hazardous substances, the protection afforded to companion animals in these instances is not intrinsic; rather, it stems from the fact that the harm inflicted upon them serves as evidence that the defendant’s conduct posed a broader threat to public security.

First, in Chinese criminal judicial practice, the criteria for prosecuting drunk-driving-related dangerous driving are relatively singular, primarily requiring a blood alcohol concentration (BAC) of 80 mg/100 mL or 150 mg/100 mL [34]. In practice, intoxicated defendants typically “create trouble” while operating a motor vehicle, leading to police intervention and the subsequent discovery of their intoxication. For instance, in Case 25, the fatal collision with a companion dog served merely as the “trouble” that prompted the traffic police to test the defendant’s BAC.

Second, in traffic casualty cases involving companion animals, although courts record that the defendant’s conduct resulted in the injury or death of an animal, the decisive factor for criminal conviction remains the human casualties. Under the Interpretation of the Supreme People’s Court on Several Issues Concerning the Specific Application of Law in the Trial of Criminal Cases of Traffic Casualties, one threshold for prosecution is “causing one death with primary liability for the accident.” In Case 26, the defendant’s conduct already met this threshold; thus, the “death of the companion dog” or “vehicle damage” served as mere objective descriptions tangential to the core criminal facts.

Finally, in cases involving the illegal possession of firearms, such as Case 27, the companion animal acted only as a catalyst that exposed the defendant’s underlying criminal act of possessing illegal weapons.

#### 3.2.2. Protection of Companion Animals via Crimes Against the Order of the Socialist Market Economy

In practice, the illicit industry involving the poisoning, theft, and trafficking of dogs is primarily driven by the production and sale of dog meat. This persistent illegal phenomenon is fueled by a sustained “market demand” for dogs as food ingredients. The illegal production and sale of dog meat often trigger the Crime of Producing or Selling Poisoned or Harmful Food [21], as criminals frequently employ toxic substances—such as poisoned bait or darts—to incapacitate and steal dogs. However, this offense is categorized under Chapter III of the Chinese Criminal Law (Crimes Against the Order of the Socialist Market Economy). Its legal interest is not the protection of animals, but rather the administrative system for food safety and the life and health of the general public [24]. In other words, the focus of Chinese criminal justice remains “human-centric,” aiming to prevent human consumption of toxic food; any protection afforded to animals in such cases is merely incidental.

Furthermore, the Chinese criminal justice system intermittently provides protection against the consumption of substandard feed, the injection of counterfeit vaccines, or the use of inferior veterinary drugs. Such instances involve the Crime of Producing or Selling Fake or Substandard Products and the Crime of Producing or Selling Fake or Substandard Veterinary Drugs [21]. Nevertheless, these offenses also fall under Chapter III, protecting the state’s supervision of product quality and the legitimate rights of consumers [35]. Consequently, any protection of animal welfare remains a “reflexive interest”, secondary to the maintenance of market order. The judicial application of these principles is further illustrated in the case summaries provided in Table 7.

In Case 28, the defense challenged the determination of “toxic and harmful non-food raw materials.” For instance, Defendants J and K claimed they were unaware that the deceased dogs were toxic. Their defense counsel argued that the conduct should instead be classified as the Crime of Selling Food Not Meeting Safety Standards, contending that while some dogs were toxic, others were not. Similarly, Defendants M and L maintained they did not know the dogs they shot were poisoned. Furthermore, Defendant N argued that the dogs were merely unconscious rather than poisoned, noting that he and others had consumed the meat without ill effects.

Under Chinese criminal law and theory, the Crime of Producing or Selling Poisoned or Harmful Food is an intentional offense; the perpetrator must have subjective awareness —either knowing that they are adding toxic substances to food or knowingly selling food that contains such materials—and, while recognizing the risk of foodborne illness, still desires or allows such consequences to occur [22]. The defense based its argument precisely on the defendants’ alleged lack of knowledge regarding the toxicity of the deceased dogs.

However, Chinese criminal theory posits that this “subjective awareness” does not necessitate absolute certainty; recognizing a possibility of toxicity satisfies the legal requirement [26]. This article contends that since the defendants were fully aware that the dogs were either poisoned by their own hands or acquired through toxic means, such awareness is sufficient to establish the intent required for “toxic and harmful non-food raw materials.” Given that this offense carries severe penalties, such a determination serves as an effective deterrent against the illicit collection of dog meat.

Case 29 is recognized as one of the “Ten Typical Cases of Intellectual Property Crimes” jointly released by the Shanghai Third Intermediate People’s Court and the Shanghai People’s Procuratorate Third Branch. Its significance as a landmark case stems from the defendants’ production and sale of counterfeit brand-name veterinary drugs. Because substandard ingredients were detected in a portion of these counterfeit products, the case involved both the Crime of Counterfeiting Registered Trademarks and the Crime of Producing or Selling Fake or Substandard Products [21].

In this instance, the prosecutorial and judicial organs conducted a substantive determination of whether the veterinary drugs were “fake” by analyzing their chemical composition. This included verifying whether biological agents (vaccines) contained improper chemical additives, whether antiviral or antibacterial drugs contained illegal substances, and whether products contained the active ingredients claimed on their labels. Where products were formally non-compliant but lacked detectable illegal additives or missing labeled components—making it impossible to determine the “substantive harm”—the court adopted the principle of in dubio pro reo (favoring the defendant), convicting them of the lesser offense of counterfeiting registered trademarks [36].

Regardless of the final charge or the specific legal interest being protected, the enforcement of laws against the production and sale of substandard animal feed, vaccines, and drugs effectively prevents these inferior products from reaching companion animals and other livestock. Thus, by punishing market-order offenses, the Chinese criminal justice system performs a reflexive role in safeguarding animal health and promoting overall animal protection.

#### 3.2.3. Protection of Companion Animals via Crimes Against the Order of Social Administration

In practice, some individuals may target companion animals as a means of inflicting harm or harassment upon their owners. These actions often fall under the Crime of Picking Quarrels and Provoking Trouble, which is categorized under Section 1 (Crimes of Disturbing Public Order) of Chapter VI (Crimes Against the Order of Social Administration) of the Chinese Criminal Law [21]. A selection of representative cases is provided in Table 8.

The cases identified in this study involving companion animals primarily fall into two categories: first, where actions taken against an animal serve as evidence that the defendant “intimidated others under egregious circumstances” (e.g., Case 30); and second, where such actions prove the defendant “arbitrarily damaged public or private property under serious circumstances” (e.g., Case 31). Furthermore, only a small number of cases involve offenses directed exclusively at companion animals (e.g., Case 32). In most instances, the harm to the animal is merely “incidental” to the defendant’s broader disruptive conduct (e.g., Case 33). Within these legal frameworks, companion animals are protected only as the peripheral property or “attachments” of the human victim.

## 4. Gaps and Improvements in the Criminal Judicial Protection of Companion Animals in China

### 4.1. Gaps in the Criminal Judicial Protection of Companion Animals

The glaring gap in China’s criminal judicial protection of companion animals lies in the failure of judicial authorities to penalize the production, sale, and dissemination of animal cruelty media. In contemporary Chinese society, these activities have coalesced into a “professionalized” underground industry. While this illicit trade has drawn significant public outcry and sparked urgent calls for legal accountability, criminal prosecution of such acts remains entirely absent.

China Comment, a Chinese journal, exposed the dark reality of “cat abuse videos.” By late 2020, their investigation revealed a sophisticated underground industry dedicated to producing, selling, and disseminating media documenting animal slaughter. One volunteer provided reporters with 130 GB of cat abuse footage and 60 GB of dog abuse videos. Between April and October 2020 alone, volunteers documented over 100 animal cruelty incidents across 21 provinces, featuring methods as brutal as live burial.

These videos are primarily circulated via groups on QQ, an instant messaging platform. One volunteer reported tracking over a dozen such groups, with memberships ranging from dozens to hundreds. To evade detection, these groups use coded language, adopting names like “Animal Lovers” or “Performance Art Appreciation,” and labeling graphic files as “Study Materials” or “Caring Videos.”

To maintain secrecy, group members are highly cautious: groups are often dissolved and reorganized every one to two days. Moderators conduct real-time vetting, moving the community immediately if an unfamiliar user is detected. New members must often pass “tests,” such as livestreaming an act of animal abuse while making a “V” for victory sign. Members also organize offline gatherings to torture animals collectively.

Beyond QQ, sellers use platforms like Weibo, WeChat, and Baidu Tieba to lure buyers. Transactions are completed via QR codes or payment links, with content shared through Baidu Wangpan (cloud storage) links. Prices range from 2 to 10 RMB per video, with “bundles” priced between 30 and several hundred RMB. Standard rates are approximately 40 RMB for 200 videos, while “original” content can cost 1 RMB per minute. According to a volunteer who went undercover, most animals are purchased specifically for these acts. The demographic of this trade includes those seeking profit through resale, individuals seeking sadistic gratification, and the merely curious. Alarmingly, participants are increasingly younger, with a significant number of minors now involved [37].

Reporters from Legal Daily, a national legal newspaper, conducted an undercover investigation into various social media groups. Their findings revealed a price hierarchy in animal cruelty media: footage involving rodents is relatively inexpensive, while media documenting the torture of cats and dogs commands higher prices. The animals used are sourced through diverse channels, including the trapping of strays, theft, and purchases from cat and dog traffickers, legitimate pet markets, breeding farms, and private owners.

A complete industrial chain has emerged, spanning from the acquisition of pets to the filming and subsequent sale of torture media. The groups infiltrated by Legal Daily reporters primarily represent the mid-to-downstream segments—specifically the execution and filming of the abuse. While many of these groups require a referral from an acquaintance to join, some remain open to the public. These open groups occasionally post “video advertisements” disguised as pet-friendly content to lure animal lovers; the perpetrators do so with the deliberate intent to harass and provoke those who care for animals [38].

Further investigations by Legal Daily uncovered “livestreamed abuse” sessions where perpetrators broadcast their acts in real-time. These rooms often feature “tipping” or “reward” systems, allowing viewers to pay to “customize” the specific methods of torture used on kittens. Beyond the sale of videos, some individuals exploit the lives of small animals for extortion. For instance, perpetrators have been known to “adopt” stray cats under false pretenses, only to demand ransoms from the original rescuers. In one case, a rescuer was extorted for over 1000 RMB; upon refusing the payment, they received a video of the cat being tortured the following day [39].

The underground industry involving the production, sale, and dissemination of animal torture media has drawn the attention of several deputies to the National People’s Congress (NPC), leading to formal proposals during NPC sessions. For instance, some deputies have called for comprehensive anti-cruelty legislation while advocating for stricter oversight of pet breeding and sales to sever the supply chain of this underground trade. Others, noting the increasing involvement of minors as perpetrators and distributors, have proposed integrating animal welfare and “life education” into the primary and secondary school curricula [40].

In practice, the participants in this illicit industry include not only minors but also university students. Several high-profile cases have triggered significant public outcry and subsequent institutional discipline. For example, a university in Jiangxi Province expelled a student after an investigation confirmed that he had repeatedly abused a pet cat in his dormitory and committed other disciplinary violations [41]. Similarly, a university in Henan Province expelled a student for abusing cats, posting the footage online, and making inappropriate public statements [42].

Furthermore, social accountability has begun to influence academic and professional prospects. A candidate who ranked first in the written component of a Master’s entrance examination at a university in Jiangsu Province was ultimately rejected during the interview stage, reportedly due to public reports of his prior involvement in filming cat abuse and participating in online animal cruelty rings [43]. In another instance, a candidate for a public institution position in Guangxi passed both written and oral examinations but was disqualified during the background check. It was discovered that he was the same individual previously issued a severe warning by a university in Hubei for cat abuse; local human resources authorities subsequently declared him unfit for recruitment [44].

While the involvement of minors and students in these activities is deeply distressing, they represent only a fraction of those operating within this rampant underground industry. Despite the professionalization and audacity of these criminal networks, China’s current criminal law has failed to provide a sufficient deterrent response. The following sections will explore potential charges within the existing Chinese criminal framework that could be utilized to regulate the production, sale, and dissemination of animal torture media.

### 4.2. Potential Charges Within the Framework of China’s Current Criminal Law

This article argues that several specific offenses within the Chinese Criminal Law—namely, the crime of illegal use of information networks and the crime of refusing to fulfill information network security management obligations [21]—could be effectively utilized to regulate the production, sale, and dissemination of animal torture media.

#### 4.2.1. The Crime of Illegal Use of Information Networks

This article argues that the crime of illegal use of information networks—stipulated in Article 287a of the Chinese Criminal Law—can be effectively applied to curb animal torture media. The basis for this argument is that such media constitutes “prohibited items” or “other information concerning illegal or criminal acts” as defined under Paragraph 1, Items (1) and (2) of this article.

This perspective is corroborated by several administrative and security regulations. For instance, Article 13 of the Cybersecurity Law of the PRC mandates that internet users must respect social morality and public order, prohibiting the dissemination of violent or pornographic information. Furthermore, the Administrative Measures on Internet Information Services (Articles 15 and 20) and the Provisions on the Ecological Governance of Network Information Content (Articles 7 and 19) explicitly prohibit or require the prevention of content that depicts bloodiness, cruelty, or horror—specifically materials that cause physical or mental distress or might induce harmful behaviors in minors.

Based on these regulations, animal torture media is not merely a violation of social ethics; it is categorized as “violent information” and “harmful content” due to its cruel and bloody nature. Consequently, such media qualifies as both illegal information and prohibited items. Therefore, the production, sale, and dissemination of such content can be interpreted as “publishing information concerning the production or sale of prohibited items” under Article 287a, Paragraph 1, Item (2). Moreover, establishing dedicated websites, QQ groups, or WeChat groups to facilitate the viewing or trading of these materials aligns with Item (1) of the same paragraph, which penalizes “establishing websites or communication groups for the production or sale of prohibited items.”

However, a potential practical obstacle remains. According to Article 7 of the Judicial Interpretation issued by the Supreme People’s Court and the Supreme People’s Procuratorate, “illegal or criminal acts” in this context refer to crimes or acts that align with the types of offenses defined in the Criminal Law but do not yet meet the threshold for criminal prosecution [45]. Some may question whether animal torture itself—currently not a standalone crime in China—fits this definition [46].

To clarify, this challenge can be resolved through a dual-track approach. First, as established above, because animal torture media independently qualifies as a “prohibited item” under administrative regulations, penalizing its dissemination or the establishment of communication groups for its sale does not legally depend on the existence of a standalone animal cruelty offense. Second, even if evaluated under the “illegal or criminal acts” track, an indirect legal approach remains viable: when the tortured animals are obtained through theft or the act involves the intentional destruction of owned animals, these actions constitute property offenses that “do not yet meet the threshold for criminal prosecution.” Therefore, under either track, the statutory requirements of Article 287a are satisfied.

#### 4.2.2. The Crime of Refusing to Fulfill Information Network Security Management Obligations

This article argues that the crime of refusing to fulfill information network security management obligations—stipulated in Article 286a of the Chinese Criminal Law—plays a crucial role in curbing animal torture media. This argument is based on two premises: first, Article 286a, Paragraph 1, Item (1) specifically penalizes failures that “result in the widespread dissemination of illegal information”; second, existing legal frameworks mandate that network service providers prevent the dissemination of such illegal content.

Specifically, Article 49 of the Cybersecurity Law of the PRC requires network operators to strengthen the management of user-published information. Upon discovering information prohibited by law or administrative regulations, they must immediately stop its transmission, take remedial measures to prevent its spread, preserve relevant records, and report to competent authorities. Violations of these duties are subject to administrative penalties under Article 69 and may lead to criminal prosecution under Article 76 if they constitute a crime. Similarly, Articles 10 and 35 of the Provisions on the Ecological Governance of Network Information Content reinforce these obligations, requiring platforms to resist harmful information and subjecting them to legal liabilities for failing to do so.

If network operators and content service platforms fulfill these duties upon detecting animal torture media, the unchecked dissemination of such content can be effectively blocked. Conversely, failure to implement these measures may constitute the crime of refusing to fulfill information network security management obligations. It is important to note, however, that a prerequisite for this offense is the “refusal to rectify after being ordered to do so by regulatory authorities.” Therefore, the effective application of this charge to curb animal torture media also necessitates proactive oversight and the diligent fulfillment of duties by the relevant regulatory bodies.

## 5. The Indirect Nature of Criminal Judicial Protection for Companion Animals in China

The criminal judicial protection of companion animals in China is characterized by a distinct indirectness, which manifests in two primary aspects.

First, as analyzed in Section 3, all current judicial cases demonstrating a protective tendency toward companion animals do so only incidentally or reflectively. This protection is achieved through offenses designed to safeguard either private individual interests or public social interests. In some cases where companion animals are “reflectively” protected through public interest offenses, the claim of “animal protection” can even seem tenuous. In these instances, defendants are convicted not because they harmed animals, but because the animals “happened” to be part of the factual record—serving as evidence of drunk driving, traffic casualties, illegal possession of firearms, concealing criminal gains, producing harmful food, or “picking quarrels and provoking trouble.” Simply put, the harm inflicted on animals serves merely as auxiliary evidence to establish the core facts of the crime.

By contrast, the protective element appears less tenuous in cases involving intentional destruction of property, theft, or releasing hazardous substances. In these scenarios, the defendants’ actions are directly targeted at the animals. However, these offenses remain, at their core, instruments to protect human interests. The decisive judicial reasoning consistently centers on logic such as “a pet with monetary value belonging to a victim was poisoned or stolen,” or “the defendant released hazardous substances in a public area, and although only animals were killed, there remained a significant risk of accidental human exposure or ingestion.”

This “indirect” logic is not unique to China but reflects a systemic “accountability gap” in global justice. As underscored in the World Animal Justice (WAJ) Report (2025), modern legal frameworks remain fundamentally “anthropocentric,” treating animals as mere “property” or “objects of ownership” [47]. Consequently, any criminal protection afforded to them is often “incidental” or “indirect,” triggered only when their harm intersects with human interests or public safety.

This systemic gap is empirically verified by the evolution of companion animal legislation and social attitudes across Western legal traditions. Historically, early statutory protections for companion animals were overtly tethered to public decency rather than animal suffering; for instance, the French Grammont Law of 1850 criminalized animal cruelty exclusively when committed “in public”. Even the celebrated modern civil code reforms in Europe—where nations like Austria, Germany, Switzerland, and the Czech Republic declared that “animals are not objects” —consistently yield to an inherent paradox. As exemplified by Article 494 of the Czech Civil Code, such progressive codifications frequently retain statutory mechanisms that allow property laws to remain residually applicable to animals unless specific exceptions dictate otherwise [48]. Consequently, in private law domains such as tortious damages, succession, or ownership disputes, companion animals are still treated as functional legal property. These realities demonstrate that even under progressive legislation, the legal safety net for companion animals remains predominantly indirect rather than rights-based.

Second, the offenses proposed in Section 4 of this article can also only provide indirect protection. The acts this article suggests penalizing—such as the production, sale, and dissemination of animal torture media—are “secondary processing” behaviors of the primary acts of animal cruelty. Furthermore, within the framework of the Chinese Criminal Law, the crime of illegal use of information networks and the crime of refusing to fulfill information network security management obligations fall under Chapter 6 (Crimes Against the Order of Social Administration). Both chapters are fundamentally dedicated to protecting the public interests of “society” rather than the inherent rights of the animals themselves.

Similarly, the United Kingdom exhibits a bifurcated approach. While the Animal Welfare Act 2006 directly penalizes physical acts of cruelty, the modern battle against digital animal torture media is instead waged through the Online Safety Act 2023. Under this framework, the UK Government formally listed Section 4(1) (unnecessary suffering) of the Animal Welfare Act 2006 as a “priority offence” within the modern internet legislation [49]. This statutory integration legally compels tech platforms to proactively detect and remove animal torture content as a strict category of “illegal content.”

This persistent indirectness underscores a deeper jurisdictional crisis. As Todorović points out, animals in most legal systems are currently trapped in a “grey zone,” functioning as “non-thing things”—entities that are theoretically recognized as non-objects but are still treated as legal objects or property in practice. Unless the legal paradigm shifts from a nature-based approach to a “sentience-based approach” that recognizes animals as independent “interest-holders,” any criminal protection will remain a secondary byproduct of safeguarding human interests [50].

## 6. Conclusions

In the persistent absence of specialized legislation for the protection of companion animals, a significant legislative vacuum remains in China. Within the current framework of the Chinese Criminal Law, judicial authorities have addressed egregious acts against animals primarily through two avenues: offenses against private interests, such as intentional destruction of property and theft, or offenses against public interests, such as releasing hazardous substances and producing or selling toxic or harmful food. Although these are fundamentally “human-centric” crimes—rendering their protection of companion animals inevitably indirect—they currently represent the most potent legal instruments available to the Chinese judiciary in the absence of a standalone crime of animal cruelty.

However, a more comprehensive judicial response has yet to be fully realized. The underground industry involved in the production, sale, and dissemination of animal torture media remains largely outside the current regulatory reach of the criminal legal system. If judicial authorities can fully tap into the potential of existing charges—namely the crime of illegal use of information networks and refusing to fulfill information network security management obligations—the protection of companion animals in China would be strengthened. On the one hand, individuals who disseminate animal torture media online or establish related communication groups and websites may constitute the crime of illegal use of information networks, as such media can be interpreted as “prohibited items” or “information concerning illegal acts.” On the other hand, network service providers are obligated to prevent the spread of animal torture media upon discovery; failure to fulfill this duty—specifically, refusing to rectify after being ordered by regulatory authorities, thereby resulting in widespread dissemination—may trigger criminal liability for the crime of refusing to fulfill information network security management obligations.

Nevertheless, regardless of how rigorously one applies interpretive theories, the underlying legislative deficiency remains an undeniable reality. This is because invoking cybercrime statutes, while serving as a practical strategy, fundamentally treats animal torture media as an offense against cyber-order rather than an offense against animal life itself. Consequently, this legal mechanism serves as a reflective remedy that inevitably leaves the intrinsic value of animals unprotected.

Ultimately, it must be recognized that criminal law serves only as the baseline for animal protection. While this article strives to explore the untapped potential of current criminal statutes through legal interpretation, a fundamental improvement in animal welfare depends on the reconstruction of the relationship between humans and animals within social ethics. The coercive power of the law should be integrated with systematic life education, facilitating a shift in public perception from viewing animals as mere “legal objects” to recognizing their intrinsic value as “sentient beings.” This cognitive transition—from “instrumental protection” to “life-oriented protection”—is not only the ethical foundation for China’s future specialized animal protection legislation but also an essential path toward ecological justice and the harmonious coexistence of humanity and nature.

## Figures and Tables

**Table 1:** Criminal charges and misconduct related to companion animal harm in China.

No.	Specific Charge	Article No. in the Criminal Law	Chapter in the Criminal Law	Animal-Related Misconduct
1	Releasing hazardous substances	114	Chapter II Crimes of Endangering Public Security	Poisoning causing death of another’s companion dog.
2	Illegal possession of firearms	128	Chapter II Crimes of Endangering Public Security	Using an illegally possessed firearm to injure or kill another person’s companion animal.
3	Crime of causing traffic casualties	133	Chapter II Crimes of Endangering Public Security	Drunk driving causing deaths of a victim and their companion dog.
4	Dangerous driving	133a	Chapter II Crimes of Endangering Public Security	Drunk driving running over another’s companion dog.
5	Producing or selling fake or substandard products	140	Chapter III Crimes of Undermining Order of Socialist Market Economy	Production or sale of fake or substandard companion animal supplies, vaccines, or medicines.
6	Producing or selling toxic or harmful food	144	Chapter III Crimes of Undermining Order of Socialist Market Economy	Production or sale of food using poisoned dogs as raw ingredients.
7	Producing or selling fake or substandard veterinary medicines	147	Chapter III Crimes of Undermining Order of Socialist Market Economy	Production or sale of fake or substandard companion animal vaccines or medicines.
8	Smuggling rare animals	151	Chapter III Crimes of Undermining Order of Socialist Market Economy	Smuggling driven by market demand for keeping these animals as companion animals.
9	Robbery	263 and 269	Chapter V Crimes Against Property	Robbery: Resorting to violence or coercion upon discovery during a dog theft to suppress the victim’s resistance, thereby securing unlawful possession of the companion dog. Transformed Robbery: Employing violence or immediate threats of violence on the spot after successfully taking the companion dog to resist capture or retain the property.
10	Theft	264	Chapter V Crimes Against Property	Theft of companion animals (including dogs, cats, hamsters, rabbits, pigeons, parrots, ducks, and snakes), predominantly involving dogs stolen by professional thieves for commercial sale.
11	Forcible seizure	267	Chapter V Crimes Against Property	Forcible seizure of the companion dog.
12	Embezzlement	270	Chapter V Crimes Against Property	Taking care of another person’s companion animal and subsequently refusing to return it.
13	Extortion	274	Chapter V Crimes Against Property	Extortion of a companion animal owner by threatening to kill their companion animal.
14	Intentional destruction of property	275	Chapter V Crimes Against Property	Poisoning a dog beforehand with the intent of theft; Poisoning a dog for the purpose of retaliating against the owner.
15	Crime of Picking Quarrels and Provoking Trouble	293	Chapter VI Crimes of Obstructing Administration of Public Order	Killing another person’s dog without cause.
16	Concealing or disguising criminal gains	312	Chapter VI Crimes of Obstructing Administration of Public Order	Knowingly purchasing stolen dogs from others.
17	Endangering precious or endangered wild animals	341	Chapter VI Crimes of Obstructing Administration of Public Order	Illegal hunting, acquisition, transportation, and sale driven by market demands such as keeping these animals as pets.

**Table 2:** Case summaries of companion animal protection via the charge of intentional destruction of property.

Case No.	Case Summary
Case 1	Five defendants used tools including hay cutters and shovels to damage windows, appliances, furniture, and glassware at the victim’s residence and killed one companion dog. The appraised value of the destroyed property was 7039 RMB (approximately 985 USD).
Case 2	Four defendants, armed with knives and iron bars, damaged the windows, potted plants, kitchen, and staff vehicles parked nearby at the victim organisation’s office; they also killed a companion dog. The appraised repair cost for the damaged vehicles alone was 24,200 RMB (approximately 3388 USD).
Case 3	The defendant destroyed the victim’s television and mobile phone and killed a companion dog by throwing it to the ground. According to the appraisal, the loss value of the destroyed television was 2295 RMB, and the loss value of the mobile phone was 3800 RMB (approximately 532 USD).
Case 4	Out of resentment over the disturbance caused by the victim’s companion dogs, the defendant placed pesticides into the dogs’ drinking water bucket, resulting in the death of six dogs by poisoning. According to the appraisal, the value of the poisoned dogs was 23,500 RMB (approximately 3290 USD).
Case 5	With the intent to consume dog meat, four defendants attempted to steal dogs by poisoning them. They threw toxic bait to the victim’s dog; however, after picking up the dog that had consumed the bait and collapsed, they abandoned it upon being discovered. According to the appraisal, the value of the poisoned dog was 8000 RMB (approximately 1120 USD).
Case 6	With the intent to traffic dogs, the defendant attempted to steal a dog by throwing toxic bait. The dog died immediately after consuming the bait; however, the defendant fled the scene upon being discovered. Subsequently, another dog owned by the victim also consumed the remaining bait and died instantly. According to the appraisal, the values of the two poisoned dogs were 800 RMB (approximately 112 USD) and 11,000 RMB (approximately 1540 USD), respectively.
Case 7	Four defendants used crossbows to fire poisoned darts, killing one dog belonging to Victim A, valued at 525 RMB. (approximately 73.5 USD) and another belonging to Victim B, valued at 10,000 RMB (approximately 1400 USD). However, they failed to steal the dogs due to factors beyond their control. The court held that to achieve their goal of stealing property, the defendants employed destructive means. Their conduct implicated both the crime of intentional destruction of property and the crime of theft. Following the principle of choosing the more severe penalty among concurring offences, the defendants were sentenced for the crime of intentional destruction of property.
Case 8	With the intent to consume dog meat, the defendant attempted to steal the victim’s dog by firing a poisoned dart from a crossbow. However, the theft was not completed due to factors beyond the defendant’s control. According to the appraisal, the value of the dog was 5400 RMB (approximately 756 USD).
Case 9	Two defendants attempted to steal two dogs from the victim by throwing toxic bait. Although they failed to carry out the theft, both dogs died after consuming the bait. According to the appraisal, the combined value of the two dogs was 24,100 RMB (approximately 3374 USD).

**Table 3:** Case summaries of companion animal protection via the charge of Theft.

Case No.	Case Summary
Case 10	The defendant used a crossbow to fire anesthetic darts, stealing one dog belonging to Victim H, valued at approximately 600 RMB (approximately 84 USD) and another belonging to Victim C, valued at approximately 600 RMB (approximately 84 USD). The court held that the defendant, acting with the intent of illegal possession, committed theft while carrying a deadly weapon. Accordingly, the defendant’s conduct constituted the crime of theft.
Case 11	Defendants G and Z conspired to steal domestic dogs from rural households. They carried out the thefts by firing poisoned darts from crossbows or feeding the dogs poison. On a single night, G committed two thefts and Z committed four. Additionally, G independently committed three more thefts using a crossbow and poisoned darts. According to the appraisal, the total value of the dogs stolen by G was 3022 RMB (approximately 423 USD), while those stolen by Z totaled 1075 RMB (approximately 150 USD). The court held that Defendant G, acting with the intent of illegal possession, committed multiple thefts while carrying a deadly weapon, involving a “relatively large amount” of property. Defendant Z, also acting with the intent of illegal possession, committed theft while carrying a deadly weapon involving a “relatively large amount.” Both defendants’ actions constituted the crime of theft.
Case 12	Defendants W and X colluded to steal dogs by shooting them with anesthetic crossbows. They transported the stolen dogs via long-distance buses to another location and sold them to merchants at a local market. The gains of 13,620 RMB (approximately 1906 USD) were split equally between them. The court held that the two defendants, acting with the intent of illegal possession, committed multiple thefts involving a “relatively large amount” of property. Their conduct constituted the crime of theft.
Case 13	The defendant used a crossbow to fire poisoned needles, first incapacitating and stealing a dog belonging to Victim H, then incapacitating two more dogs belonging to a victim organization but failing to carry them away. According to the appraisal, the combined value of the dogs involved was 4700 RMB (approximately 658 USD). The court held that the defendant, acting with the intent of illegal possession, committed secret misappropriation of another’s property involving a “relatively large amount.” Accordingly, the defendant’s conduct constituted the crime of theft.

**Table 4:** Case summaries of companion animal protection via other crimes against private interests.

Case No.	Case Overview	Judicial Determination
Case 14	The defendant, acting with accomplices and equipped with tools such as woven bags and bolt cutters, attempted to steal a dog from Victim L. After being caught in the act by L, the defendant brandished a wooden club to intimidate the victim and forcibly took the dog, valued at 300 RMB (approximately 42 USD).	The court held that the defendant, acting with the intent of illegal possession, employed coercive threats to forcibly seize the property after being discovered during the theft. This conduct constituted the crime of robbery.
Case 15	Taking advantage of the fact that victims A (aged 10) and B (aged 11) were vulnerable minors, the defendant openly snatched a dog, valued at 2083 RMB (approximately 291 USD), while A was walking it.	The court held that the defendant, acting with the intent of illegal possession, openly snatched property from minors. Given that the value involved was “relatively large,” the defendant’s conduct constituted the crime of forcible seizure.
Case 16	Under the pretext of discussing business, Defendant C lured Victim T to a meeting and instructed other defendants to take T to a designated location. There, the defendants extorted 11,000 RMB (approximately 1540 USD) from T by threatening to kill T’s dog if the money was not paid.	The court held that Defendant C gathered multiple other defendants to repeatedly carry out illegal and criminal activities, including extortion and fraud, within a specific region through the use of violence, threats, and other means. This group was identified as an “evil forces” crime syndicate, within which the actions taken against Victim T constituted part of their collective extortive activities.
Case 17	The private prosecutor alleged that they had entrusted the defendant with the care of a dog, valued at 30,000 RMB (approximately 4200 USD), and provided funds for its maintenance and medical treatment. Later, the defendant expressed an interest in purchasing the dog and informed the prosecutor of their intention to keep it. Despite the prosecutor’s refusal and repeated demands for the dog’s return, the defendant persisted in withholding the dog.	The court held that the evidence submitted by the private prosecutor indicated the defendant’s failure to return the dog stemmed from a dispute over unpaid maintenance fees. Consequently, the court found the facts and evidence insufficient to support the allegation of embezzlement. The private prosecutor’s filing was ultimately ruled inadmissible.
Case 18	Upon learning that Defendant D was in the dog-trading business, Defendant C recruited Defendants Z, A, and B to steal dogs for sale to D. Despite being fully aware that the dogs were stolen, D repeatedly purchased and resold them, resulting in the dogs being unrecoverable.	Judicial Determination: The court held that defendants C, Z, A, and B, acting with the intent of illegal possession, committed multiple thefts of property through secret misappropriation; their conduct constituted the crime of theft. Defendant D, despite knowing that the dogs sold by C were gains of theft, continued to purchase them; this conduct constituted the crime of concealing or disguising criminal gains.
Case 19	Defendants A and B conspired with C, agreeing that C would steal dogs while A and B would purchase and resell them. Defendant D, fully aware that the dogs were stolen by C, transported and sold a portion of the stolen dogs, valued at 6000 RMB (approximately 840 USD), to A and B.	The court held that defendants A, B, and C, acting with the intent of illegal possession and through prior conspiracy, committed multiple thefts of property involving a “relatively large amount.” Their actions constituted the crime of theft as a joint offense. Defendant D, despite knowing the dogs were criminal gains, engaged in acts of concealment; such conduct constituted the crime of concealing or disguising criminal gains.

**Table 5:** Case summaries of companion animal protection via Crimes Against Public Security.

Case No.	Case Overview	Defense Arguments	Prosecutorial and Judicial Perspectives
Case 20	Having previously been bitten by a dog, the defendant placed poisoned bait on the lawns of a residential community. This action resulted in the accidental ingestion and death of six companion dogs belonging to five different residents.	Defendant: (1) The sole intent was to poison free-ranging dogs; (2) The toxicity of the substance used was relatively low; (3) When placing the bait, there was no consideration of the consequences of accidental human consumption or the consumption of the poisoned carcasses by humans. Defense Counsel: (1) The location of the incident does not qualify as a “public space” in a legal sense; (2) The defendant lacked the subjective intent to endanger public security; (3) The conduct should be classified as the crime of intentional destruction of property rather than a crime against public security.	The court held that (1) The lawns within a residential community are collectively owned by all residents and possess characteristics of openness and mobility, thus qualifying as a “public space” in a legal context; (2) The defendant’s conduct not only infringed upon the property rights of the dog owners but was also sufficient to endanger the lives and property safety of an indiscriminate majority of people.
Case 21	Driven by resentment over the unsanitary disposal of pet waste in their residential community, Defendants A and B conspired to poison pet dogs by placing toxic bait in public areas, including community lawns. Following their agreement, A provided the poison to B, who subsequently prepared and distributed the toxic bait, resulting in the poisoning and death of 11 companion dogs.	Defense Counsel: (1) The defendants lacked the subjective intent to endanger public security. (2) The defendants’ objective conduct did not pose a genuine threat to public security based on the following: ① On the day of the incident, the inclement weather (sleet and snow) caused the poisoned bait—which had been broken into nail-sized fragments—to blend into the mud and be covered by snow upon being thrown onto the lawn, thereby precluding any possibility of accidental human pickup or consumption; ② The location where the bait was placed was a restricted area not subject to unrestricted public access. (3) The conduct should be legally classified as the crime of intentional destruction of property rather than a crime against public security.	Prosecution:(1) The sleet on the day of the offense did not impair the potency of the poison. (2) The resulting poisoning and death of 11 companion dogs demonstrably establish a significant threat to public safety. Court: (1) The defendant did not target specific animals. Any animal walking across the residential lawn could have eaten the poison and died. (2) The methods used by the defendant objectively put public safety at risk.
Case 22	Out of resentment after previously stepping on dog feces, the defendant placed poisoned bait near the entrance of a parking lot. This act resulted in the poisoning and death of seven companion dogs.	Defense Counsel: The defendant’s conduct does not constitute the crime of releasing hazardous substances. The defendant’s subjective intent was limited to the destruction of property	The court held that the area near the parking lot is a location frequently utilized by citizens and their pets for outdoor activities. Driven by personal resentment, the defendants intentionally placed toxic bait in this public space. This conduct not only resulted in the immediate death of seven large companion dogs on the day of the incident but also threatened the safety of the lives and property of an indiscriminate majority of people and animals. Therefore, the defendants’ actions constitute the crime of releasing hazardous substances.
Case 23	Motivated by a past incident where his granddaughter was knocked down by a dog, the defendant placed poisoned bait within his own villa’s courtyard as well as near the staircases of other residents’ entrances. This conduct resulted in the poisoning and death of eight companion dogs.	Defense Counsel: (1) The locations where the bait was placed lack “publicness” (legal public character) under the criminal law. (2) The act of placing the bait did not pose a substantive danger to public security. (3) The defendant lacked the subjective intent (scienter) to cause the poisoning and death of the eight companion dogs.	The court ruled that the defendant was guilty of the crime of releasing hazardous substances.
Case 24	The defendant, a property cleaner, had ongoing conflicts with residents over uncollected dog waste. As a result, the defendant placed poisoned bait on the lawns within the residential community.	Defendant: The placement of toxic substances was specifically targeted at the companion dogs of particular residents and was unlikely to cause harm to other individuals.	The court held that: (1) Lawns within a residential community qualify as public spaces; (2) The hazardous substances were small in particle size and not completely isolated from the environment, making it impossible to entirely eliminate the risk of accidental contact or ingestion; (3) Although the defendant’s act of placement had a certain degree of specificity in its target, the defendant maintained a reckless/laissez-faire attitude (indirect intent) toward other potential damages; (4) The defendant could not control the scope or actual consequences of the harm, which could lead to an imminent danger to the lives, health, or significant property of an indiscriminate majority of people.

**Table 6:** Case summaries of companion animal involvement in other Crimes Against Public Security.

Case No.	Case Overview	Judgment
Case 25	The defendant drove a motor vehicle while intoxicated and collided with an unleashed companion dog, resulting in the dog’s death. Forensic testing confirmed that the defendant’s blood alcohol concentration had met the legal threshold for drunk driving.	The court held that the defendant’s act of driving a motor vehicle on a public road while intoxicated constituted the crime of dangerous driving.
Case 26	The defendant operated a motor vehicle while intoxicated and collided with a victim who was crossing the road with a leashed companion animal. The accident resulted in the deaths of both the victim and the animal, as well as damage to the vehicle. Following the collision, the defendant abandoned the vehicle and fled the scene to conceal the fact of his impaired driving. According to the official accident liability determination, the defendant bore primary responsibility for the accident, while the victim bore secondary responsibility.	The court held that the defendant’s conduct—violating traffic transportation regulations, resulting in a fatality, and fleeing the scene—constituted the crime of causing a traffic casualty.
Case 27	Motivated by an earlier incident where his companion cat was attacked by a victim’s large dog, the defendant shot and injured the dog using a concealed firearm. Following the incident, police seized two firearms and 510 rounds of ammunition from the defendant’s possession.	The court held that the defendant’s conduct—violating firearm control regulations by illegally possessing firearms and ammunition under “serious circumstances”—constituted the crime of illegal possession of firearms and ammunition.

**Table 7:** Case summaries of companion animal involvement in Crimes Against the Order of the Socialist Market Economy.

Case No.	Case Overview	Judgment
Case 28	Defendants J and K, long-term food wholesalers, were apprehended by public security organs while acquiring deceased dogs. Authorities seized eight dog carcasses and 11 bags of frozen dog meat, which tested positive for the toxic substance suxamethonium. The supply chain involved Defendant M, who stole four local dogs by shooting them with toxic needles before selling them to J and K; Defendant M later collaborated with Defendant L to steal an additional eight dogs using toxic arrows. Testing confirmed that the liquid in the seized crossbows contained suxamethonium chloride. Furthermore, Defendant N stole over 20 dogs using toxic needles and sold them to J and K for distribution into the food market.	The court held that (1) Defendants J, K, L, M, and N knowingly sold food containing toxic and harmful non-food raw materials, which severely jeopardized public food safety and disrupted the market economic order; thus, their conduct constituted the crime of selling toxic and harmful food. (2) Regarding the dog meat already sold by J and K, the court did not uphold the charges as the public prosecution failed to provide sufficient evidence that such meat contained toxic or harmful substances. (3) Regarding the dog meat containing suxamethonium that J and K had yet to sell, the conduct was determined to be an attempted crime of selling toxic and harmful food.
Case 29	To seek illicit profits, Defendants J and S, acting individually or in collusion, engaged in the production and sale of counterfeit and substandard veterinary drugs—including the Feline 3-in-1 vaccine (FVRCP)—without the required production qualifications or authorization from trademark owners. These illicit products were distributed to the public through WeChat.	The court held that (1) To seek illicit profits, Defendants J and S decided to and directed others to produce and sell substandard veterinary drugs as qualified products; such conduct constituted the crime of producing or selling fake or substandard products. (2) Without the authorization of the trademark owners, the defendants used trademarks identical to registered trademarks on the same type of goods and sold them; such conduct constituted the crime of counterfeiting registered trademarks.

**Table 8:** Case summaries of companion animal involvement in Crime Against the Order of Social Administration.

Case No.	Case Overview
Case 30	To compel the leasing of rural land, Defendant G employed various methods to intimidate others, including carrying explosives and threatening to detonate them, using fake firearms for intimidation, and stabbing a pet with a dagger.
Case 31	To compel the relocation of victims, Defendant L directed others to throw toxic substances into the courtyards of families A, B, C, and D. This resulted in the poisoning and death of 10 dogs (two from Family A, one from Family B, six from Family C, and one from Family D), with a combined value of 5780 RMB (approximately 809 USD). Additionally, the defendant directed others to use a forklift to destroy gates, warehouses, 1000 corn seedlings, and several glass panes belonging to the victims. The court held that Defendant L directed others to arbitrarily destroy public and private property under “serious circumstances.” Such conduct constituted the crime of picking quarrels and provoking trouble.
Case 32	After being knocked down by a black dog owned by Victim B near his own residence, Defendant A pursued the dog into Victim B’s private courtyard with a shovel. There, the defendant attacked three other companion dogs belonging to B, killing two and injuring one. The value of the two deceased dogs was appraised at 2566 RMB (approximately 359 USD). The defense argued that the conduct should be classified as the intentional destruction of property, which would not meet the monetary threshold for criminal prosecution given the appraised value of 2566 RMB (approximately 359 USD). However, the court held that Defendant A’s conduct—picking a quarrel without provocation and arbitrarily destroying property under “serious circumstances”—constituted the crime of picking quarrels and provoking trouble.
Case 33	Five defendants assaulted the victim, L, at their place of business. Upon leaving, one defendant kicked and damaged a glass door and a dog crate. The crate contained a Labrador Retriever, which sustained injuries from the attack and later died despite receiving medical treatment. Forensic assessments confirmed that L sustained Category II minor injuries. The total value of the damaged property and the loss of the companion animal was appraised at 5600 RMB (approximately 784 USD). The court held that the five defendants’ unprovoked assault on the victim (resulting in minor injuries) and the prestige-motivated destruction of property, valued at 5600 RMB (approximately 784 USD), constituted “serious circumstances.” Consequently, their actions were classified as the crime of “picking quarrels and provoking trouble” under the law.

## Data Availability

All data created or analyzed in this study are available as Supplementary Material.

## Supplementary Materials

The following supporting information can be downloaded at https://www.mdpi.com/article/10.3390/ani16142119/s1, Supplementary Material: Criminal Judgments. This document contains the full text of the criminal judgments analyzed in this study (originally in Chinese), organized by their case reference numbers as cited in the main text.
