# Supplementary material for: Protecting Companion Animals Under Chinese Criminal Law: Current Practice and Future Paths
Source: Animals (Basel). 2026 Jul 8;16(14):2119. doi: 10.3390/ani16142119 (PMC13405461; doi:10.3390/ani16142119)
Supplement: Supplementary file 1 [file animals-16-02119-s001.zip › animals-4321148-supplementary/animals-4321148-supplementary7.3/Criminal Judgment of Case 1.pdf]

案例 1 刑事判决书

案　　　　由：侵犯财产罪/故意毁坏财物罪

案情：2016 年 8 月 10 日 17 时许，被告人张某、程某、李某、龚某、高某等人在某饭店被涂某（另案处理）开枪射击。当日 18 时许，张某带领程某、李某、龚某、高某等人驾车到涂某住处寻找涂某未果后，使用铡刀、铁锹等工具将涂某和郭某住处的门窗、家电、桌椅、玻璃等物品砸坏，并将一只宠物狗砍死，随后逃离现场。经鉴定，被毁坏财物价值人民币 7039 元。

判决：被告人张某、程某、李某、龚某、高某共同故意毁坏他人财物，数额较大，其行为均已构成故意毁坏财物罪。

一、对于被告人张某，判处有期徒刑七个月。

二、对于被告人程某，判处有期徒刑六个月。

三、对于被告人李某，判处有期徒刑七个月。

四、对于被告人龚某，判处拘役五个月，缓刑十个月。

五、对于被告人高某，判处拘役五个月，缓刑十个月。
