# Supplementary material for: Protecting Companion Animals Under Chinese Criminal Law: Current Practice and Future Paths
Source: Animals (Basel). 2026 Jul 8;16(14):2119. doi: 10.3390/ani16142119 (PMC13405461; doi:10.3390/ani16142119)
Supplement: Supplementary file 1 [file animals-16-02119-s001.zip › animals-4321148-supplementary/animals-4321148-supplementary7.3/Criminal Judgment of Case 10.pdf]

案例 10 刑事判决书

案　　　　由：侵犯财产罪/盗窃罪

案情：2024 年 10 月 24 日 19 时 40 分许，被告人陈某驾驶摩托车在被害人刘某家门前，通过使用其随身携带的弓弩发射麻醉针镖的方式，盗得刘某的黄灰色土狗一只，该被盗土狗重量约 30 斤，价值约 600 元。 2024 年 10 月 29 日凌晨 1 时 40 分许，被告人陈某以同样的方式，在被害人陈某经营的某店门前，盗得陈某的白色宠物狗一只，该被盗宠物狗价值约 600 元。

判决：被告人陈某以非法占有为目的，携带凶器窃取他人财物，其行为构成盗窃罪；判处有期徒刑七个月，并处罚金人民币 2000 元。
