# Supplementary material for: Protecting Companion Animals Under Chinese Criminal Law: Current Practice and Future Paths
Source: Animals (Basel). 2026 Jul 8;16(14):2119. doi: 10.3390/ani16142119 (PMC13405461; doi:10.3390/ani16142119)
Supplement: Supplementary file 1 [file animals-16-02119-s001.zip › animals-4321148-supplementary/animals-4321148-supplementary7.3/Criminal Judgment of Case 11.pdf]

案例 11 刑事判决书

案　　　　　由：侵犯财产罪/盗窃罪

案情：2024 年 9 月 2 日晚，被告人高某、张某商议结伴偷狗，各自采取弓弩发射毒镖或投喂毒药的方式盗窃村民家养土狗、宠物狗。当晚高某共作案 2 起，张某共作案 4 起。2024 年 6、7 月份，高某采取弓弩发射毒镖的方式盗窃村民家养土狗，共作案 3 起。经价格认证中心认定，高某盗窃的土狗、宠物狗价值共计 3022 元人民币，张某盗窃的土狗价值共计 1075 元。

判决：被告人高某以非法占有为目的，多次携带凶器秘密窃取他人财物，数额较大，被告人张某以非法占有为目的，携带凶器秘密窃取他人财物，数额较大，均构成盗窃罪。

一、对于被告人高某，判处有期徒刑六个月，并处罚金人民币三千元；

二、对于被告人张某，判处有期徒刑六个月，并处罚金人民币二千元。

三、扣押在案的被告人高某、张某的作案工具弩一把、毒镖、狗药均予以没收。
