# Supplementary material for: Protecting Companion Animals Under Chinese Criminal Law: Current Practice and Future Paths
Source: Animals (Basel). 2026 Jul 8;16(14):2119. doi: 10.3390/ani16142119 (PMC13405461; doi:10.3390/ani16142119)
Supplement: Supplementary file 1 [file animals-16-02119-s001.zip › animals-4321148-supplementary/animals-4321148-supplementary7.3/Criminal Judgment of Case 12.pdf]

案例 12 刑事判决书

案　　　　由：侵犯财产罪/盗窃罪

案情：被告人万某、徐某结伙流窜作案，以“麻醉弓弩射杀狗”的方式偷狗。为此，二人分工合作，由徐某出面合伙租赁车辆并套牌驾驶，万某负责在网上购买弓弩及麻醉针，使用麻醉弓弩射杀狗并销赃，所盗死狗通过班车托运到某地，以 12-13 元 / 斤的价格出售给商贩（另案处理），销售所得扣除费用后二人平分。期间，二人共获利 13620 元，各分得 6810 元。

判决：被告人徐某、万某以非法占有为目的，多次窃取他人财物，数额较大，其行为构成盗窃罪。

一、对于被告人徐某，判处有期徒刑十个月，并处罚金人民币二千元。

二、对于被告人万某，判处有期徒刑七个月，并处罚金人民币二千元。

三、徐某、万某的违法所得人民币 4810 元，予以没收，上缴国库。
