# Supplementary material for: Protecting Companion Animals Under Chinese Criminal Law: Current Practice and Future Paths
Source: Animals (Basel). 2026 Jul 8;16(14):2119. doi: 10.3390/ani16142119 (PMC13405461; doi:10.3390/ani16142119)
Supplement: Supplementary file 1 [file animals-16-02119-s001.zip › animals-4321148-supplementary/animals-4321148-supplementary7.3/Criminal Judgment of Case 13.pdf]

案例13刑事判决书

案　　　　由：侵犯财产罪/盗窃罪

案情：2023年9月7日早上7时许，被告人揭某骑摩托车窜至某地，用弓弩向被害人黄某家一只白色宠物狗（白色的萨摩耶品种）发射毒针，将狗放倒后盗走。2023年9月17日上午8时许，被告人揭某窜至某公司准备盗窃土狗，同样用发射毒针的方式将两条土狗放倒后，在被告人准备去捡时被人发现，被告人逃离作案现场。经价格认定中心鉴定，上述被盗狗的价值共为人民币4700元。

判决：被告人揭某以非法占有为目的，秘密窃取他人财物价值人民币 4700 元，数额较大，其行为构成盗窃罪；判处拘役三个月，并处罚金人民币1000元。
