# Supplementary material for: Protecting Companion Animals Under Chinese Criminal Law: Current Practice and Future Paths
Source: Animals (Basel). 2026 Jul 8;16(14):2119. doi: 10.3390/ani16142119 (PMC13405461; doi:10.3390/ani16142119)
Supplement: Supplementary file 1 [file animals-16-02119-s001.zip › animals-4321148-supplementary/animals-4321148-supplementary7.3/Criminal Judgment of Case 14.pdf]

案例14 刑事判决书

案　　　　由： 侵犯财产罪/抢劫罪
侵犯财产罪/盗窃罪

案情：

一、抢劫事实

2013 年 11 月 29 日凌晨 4 时许，被告人张某与同案人杨某、赖某（另案处理）经预谋盗窃后，驾驶 2 辆无牌摩托车，携带编织袋、省力钳等工具，到被害人李某家门口，准备盗走其家 1 只狗，被李某发现后，赖某持木棍对李某威吓后，抢走李某家狗 1 只（价值 300 元）。

二、盗窃事实

（一）2013 年 10 月 14 日凌晨 3 时许，被告人张某与同案人杨某（另案处理）经预谋后，驾驶 1 辆无牌摩托车，携带编织袋等工具，到被害人黄某家门口，趁无人之机，盗走其家狗 1 只、鹅 4 只（无法估价）。

（二）2013 年 11 月初的一天凌晨 2 时许，被告人张某与同案人杨某、陈某（另案处理）经预谋后，驾驶 1 辆无牌摩托车，携带编织袋等工具，到被害人何某的养猪场，趁无人之机，盗走其米特狗 1 只（无法估价）。

（三）2013 年 11 月下旬的一天凌晨 3 时许，被告人张某与同案人杨某、陈某（另案处理）经预谋后，驾驶 1 辆无牌摩托车，携带编织袋等工具，到被害人沈某家门口，趁无人之机，盗走其家狗 2 只（价值 750 元）。

（四）2013 年 11 月 27 日 14 时许，被告人张某与杨某（另案处理）经预谋后，驾驶 1 辆无牌摩托车，携带编织袋等工具，到被害人官某家门口，趁无人之机，盗走其家狗 2 只（价值 1000 元）。

（五）2013 年 11 月 29 日凌晨 3 时许，被告人张某与杨某、赖某（另案处理）经预谋后，驾驶 2 辆无牌摩托车，携带编织袋、省力钳等工具，到被害人赖某的养猪棚，趁无人之机，盗走其家狗 1 只（价值 330 元）。

（六）2013 年 12 月 4 日凌晨 4 时许，被告人张某与杨某（另案处理）经预谋盗窃后，驾驶 1 辆无牌摩托车，携带编织袋、夹子等工具，到被害人张某家门口，趁无人之机，盗走其家宠物狗 1 只（无法估价）。

（七）2019 年 4 月 26 日凌晨 4 时许，被告人张某到被害人黄某的出租屋，乘人不备之机，撬开门锁进入出租屋内，盗窃出租屋内煤气瓶、电饭煲各 1 个、纸巾 10 卷（无法估价）。

（八）2020 年 5 月 27 日 8 时许，被告人张某到被害人晏某的出租屋，乘人不备之机，撬开门锁进入出租屋内，盗窃出租屋内电动车充电器、影碟机、遥控器各 1 个（无法估价）。

判决： 被告人张某与同案人以非法占有为目的，窃取他人财物过程中被发现后，采取暴力胁迫手段强行劫取他人财物，其行为侵犯公民的人身权利和财产权利，已构成抢劫罪。张某还以非法占有为目的，多次窃取他人财物，数额较大，其行为侵犯公民的财产权利，又已构成盗窃罪。应依法数罪并罚。被告人张

某犯抢劫罪，判处有期徒刑三年，并处罚金人民币三千元；犯盗窃罪，判处有期徒刑一年六个月，并处罚金人民币三千元。总和刑期有期徒刑四年六个月，并处罚金人民币六千元；决定执行有期徒刑四年三个月，并处罚金人民币六千元。
