# Supplementary material for: Protecting Companion Animals Under Chinese Criminal Law: Current Practice and Future Paths
Source: Animals (Basel). 2026 Jul 8;16(14):2119. doi: 10.3390/ani16142119 (PMC13405461; doi:10.3390/ani16142119)
Supplement: Supplementary file 1 [file animals-16-02119-s001.zip › animals-4321148-supplementary/animals-4321148-supplementary7.3/Criminal Judgment of Case 15.pdf]

案例 15 刑事判决书

案　　　　　由：侵犯财产罪/抢夺罪

案情：2020 年 8 月 18 日晚 8 点 30 分许，被告人孙某在某超市门口，以被害人袁某 1（2010 年 1 月 20 日生）和袁某 2（2009 年 6 月 4 日生）系未成年人，年幼可欺，公然夺取被害人袁某 1 遛狗时的一条宠物犬，价值人民币 2083 元。

判决：被告人孙某以非法占有为目的，公然夺取未成年人财物，数额较大，其行为已构成抢夺罪；判处拘役五个月，缓刑五个月，罚金人民币二千元。
