# Supplementary material for: Protecting Companion Animals Under Chinese Criminal Law: Current Practice and Future Paths
Source: Animals (Basel). 2026 Jul 8;16(14):2119. doi: 10.3390/ani16142119 (PMC13405461; doi:10.3390/ani16142119)
Supplement: Supplementary file 1 [file animals-16-02119-s001.zip › animals-4321148-supplementary/animals-4321148-supplementary7.3/Criminal Judgment of Case 16.pdf]

案例16刑事判决书

案　　　　由：侵犯财产罪/敲诈勒索罪
侵犯财产罪/诈骗罪

案情：

2017 年 12 月至 2018 年 4 月期间，被告人陈某纠集被告人张某、周某等人以暴力、威胁或者其他手段，在一定区域内多次实施敲诈勒索、诈骗等违法犯罪活动，为非作恶，欺压百姓，扰乱经济、社会生活秩序，造成了较为恶劣的社会影响，三人在纠集期间，已形成恶势力犯罪集团。具体事实：

一、敲诈勒索

1、2017 年 12 月，被害人倪某通过被告人周某认识被告人陈某并向其借款人民币 2 万元。借款次日，被告人周某在被告人陈某的授意下，通过发送威胁微信、视频的方式向被害人倪某施加压力，催要还款。一周后被害人倪某将连本带息共计 2.7 万元通过被告人周某予以归还。

被告人周某得到上述还款后，与被告人陈某、张某共谋，以还款丢失为由，继续向被害人倪某索要该笔借款，被害人倪某因害怕受到被告人陈某等人的威胁，于 2018 年 1 月向被告人陈某借款 3.5 万元用于二次归还首次借款，2018 年 2 月，被害人倪某归还 3.5 万元给被告人陈某。

2、2018 年 1 月，被告人陈某、张某以投资开店为名，让被害人冯某甲交付 2 万元，遭到被害人的拒绝。后被告人陈某、张某采用酒瓶砸头、拳击等方式对被害人冯某甲实施敲诈勒索。被害人冯某甲向其父亲冯某乙索要 2 万元交给被告人陈某。

3、2018 年 3 月 4 日，杜某驾驶的货车与李某驾驶的轿车发生交通事故，李某请田某出面处理事故的赔偿问题，田某就请陈某等人出面处理事故，田某和陈某等人到杜某的单位与杜某商谈处理事故的问题，2018 年 4 月 19 日，杜某以 6 万元购买了李某的轿车，田某支付给陈某等人出场费 13000 元。被告人陈某认为田某给的出场费不到位，于 2018 年 4 月 23 日晚，纠集被告人张某、周某预谋对被害人田某实施敲诈勒索，并同时纠集被告人于某、张某、贾某等人帮助共同索要。2018 年 4 月 24 日 13 时许，被告人陈某以商量事情为由约谈被害人田某，并指使被告人周某、张某、贾某将被害人带至某地。被告人周某、张某、于某、张某、贾某、臧某以不给钱就杀死被害人所养宠物狗等言语威胁方式逼迫被害人田某，向其勒索财物 11000 元。事后，被告人张某、周某各分得好处费 600 元，被告人于某、张某、贾某、臧某各分得好处费人 500 元。

二、诈骗

2017 年 12 月，被告人陈某、张某与冯某甲之间没有债权债务关系，被告人陈某在没有投资开店的情况下，为了骗取他人钱财，跟冯某甲谎称要投资开店赚钱，要冯某甲投资，但冯某甲没有钱，陈某就提出要冯某甲向他家里人要，因担心冯某甲的家里人不给，被告人陈某、张某与冯某甲商议，虚构被告人陈某帮助冯某甲归还了 3 万元债务的事实，随后，被告人陈某、张某与冯某甲到冯某甲的家里以此为由要钱，冯某甲父母和爷爷给了 3 万元给陈某。冯某甲未从中分得钱财。

另查明，2018 年 11 月 27 日 19 时许，杨某（另案处理）为向被害人严某索取债务，邀约被告人张某、熊某（另案处理）等人限制被害人严某人身自由至 2018 年 11 月 28 日凌晨。期间，被告人张某对被害人严某实施辱骂、殴打行为。后被害人严某被杨某等人带至其他地点继续限制人身自由至 2018 年 11 月 28 日 13 时许。后被害人严某因其亲属报警被警察解救。

判决：被告人陈某、张某诈骗他人财物，数额较大，其行为已构成诈骗罪。被告人陈某、张某、周某、于某、张某、贾某、臧某敲诈勒索他人财物，其中被告人陈某、张某数额巨大，被告人周某、于某、张某、贾某、臧某数额较大，其行为均已构成敲诈勒索罪。被告人张某与他人共同非法剥夺他人人身自由，其行为已构成非法拘禁罪。被告人陈某、张某、张某犯数罪，依法应当数罪并罚。被告人陈某、张某、周某、于某、张某、贾某、臧某共同故意犯罪，系共同犯罪。被告人张某与他人共同犯罪，系共同犯罪。被告人陈某、张某、周某经常纠集在一起，以暴力、威胁等非法手段，在一定区域内多次实施违法犯罪活动，为非作恶，欺压百姓，扰乱经济、社会生活秩序，造成了较为恶劣的社会影响，属于恶势力。被告人陈某、张某、周某为共同实施犯罪而组成较为固定的犯罪组织，系恶势力犯罪集团。被告人陈某组织、领导恶势力犯罪集团进行犯罪活动，系主犯，且系首要分子，依法应当按照集团所犯的全部罪行处罚。被告人张某、周某在共同犯罪中起主要作用，系主犯，应当按照其所参与的全部犯罪处罚。被告人于某在敲诈勒索犯罪中起主要作用，系主犯，应当按照其所参与的全部犯罪处罚。被告人张某、贾某、臧某在敲诈勒索犯罪中起次要作用，系从犯，依法应当从轻处罚。

一、被告人陈某犯诈骗罪，判处有期徒刑一年六个月，并处罚金人民币二万元；犯敲诈勒索罪，判处有期徒刑三年三个月，并处罚金人民币三万元；数罪并罚，决定执行有期徒刑四年，并处罚金人民币五万元。

二、被告人张某犯诈骗罪，判处有期徒刑一年六个月，并处罚金人民币二万元；犯敲诈勒索罪，判处有期徒刑三年，并处罚金人民币三万元；数罪并罚，决定执行有期徒刑三年六个月，并处罚金人民币五万元。

三、被告人周某犯敲诈勒索罪，判处有期徒刑一年八个月，并处罚金人民币三万元。

四、被告人于某犯敲诈勒索罪，判处有期徒刑九个月，并处罚金人民币三千元。

五、被告人张某犯敲诈勒索罪，判处有期徒刑六个月，并处罚金人民币二千元；犯非法拘禁罪，判处有期徒刑六个月；数罪并罚，决定执行有期徒刑八个月，并处罚金人民币二千元。

六、被告人贾某犯敲诈勒索罪，判处有期徒刑六个月，并处罚金人民币二千元。

七、被告人臧某犯敲诈勒索罪，判处有期徒刑六个月，并处罚金人民币二千元。

八、被告人张某、贾某、臧某各自退出的赃款人民币五百元和被告人周某的近亲属退出的人民币三千元，发还被害人田某。

九、责令被告人陈某、张某共同退赔人民币五万元，发还被害人冯某；责令被告人陈某、张某、周某共同退赔人民币三万元五千元，发还被害人倪某；责令被告人陈某、张某、周某、于某、张某、贾某、臧某共同退赔人民币一千五百元，发还被害人田某。
