# Supplementary material for: Protecting Companion Animals Under Chinese Criminal Law: Current Practice and Future Paths
Source: Animals (Basel). 2026 Jul 8;16(14):2119. doi: 10.3390/ani16142119 (PMC13405461; doi:10.3390/ani16142119)
Supplement: Supplementary file 1 [file animals-16-02119-s001.zip › animals-4321148-supplementary/animals-4321148-supplementary7.3/Criminal Judgment of Case 17.pdf]

案例 17 刑事裁定书

案　　　　由：侵犯财产罪/侵占罪

案情：2017 年 6 月 14 日本院收到自诉人刘某的自诉状，6 月 20 日通知其补正材料，6 月 21 日提交补正材料。自诉人刘某诉称，自诉人与被告人龙某均在加拿大多伦多留学，2017 年 4 月 6 日自诉人因事回国，委托他人将自己的爱犬约克夏（价值人民币 30000 元）交给被告人照看，并转给被告人 450 加币作为狗的生活费用。同年 4 月底被告人告知自诉人不想返还，称其想购买此狗被自诉人拒绝。之后被告人称狗的腿骨折要给狗看病花费 400 加币，未在提起购买狗之事，期间还催促自诉人尽快将狗带走。同年 5 月中旬，自诉人再次向被告人打款 500 加币作为狗的复查费用，被告人再次提出要将狗占为己有，遭自诉人拒绝后被告人自行决定将狗带回国。期间自诉人多次和被告人交涉要求返还狗，被告人拒不返还并拒收自诉人转账 700 加币。自诉人无奈报案，民警给被告人打电话但被告人拒不返还，后自诉人又报案，民警调解未果。自诉人认为，被告人将代为保管的他人财物非法占为己有，数额较大，拒不退还，其行为已构成侵占罪，应依法追究其刑事责任；并要求判令被告人立即返还自诉人的宠物狗。

裁定：本案中自诉人提交的微信聊天记录显示，被告人未向自诉人返还宠物狗是由于双方因宠物狗的代管费用问题发生争议，现自诉人指控被告人犯侵占罪的事实和证据不足；对自诉人刘某的起诉，不予受理。
