# Supplementary material for: Protecting Companion Animals Under Chinese Criminal Law: Current Practice and Future Paths
Source: Animals (Basel). 2026 Jul 8;16(14):2119. doi: 10.3390/ani16142119 (PMC13405461; doi:10.3390/ani16142119)
Supplement: Supplementary file 1 [file animals-16-02119-s001.zip › animals-4321148-supplementary/animals-4321148-supplementary7.3/Criminal Judgment of Case 18.pdf]

# 案例 18 刑事判决书

**案　　　　由：**侵犯财产罪/盗窃罪
妨害社会管理秩序罪/妨害司法罪/掩饰、隐瞒犯罪所得、犯罪所得收益罪

**案情：**被告人程某得知被告人邓某从事贩狗生意，遂于 2015 年 10 月至 2015 年 11 月 17 日期间，被告人程某先后邀约被告人张某、姜某甲、姜某乙盗窃犬只。

1.2015 年 10 月中旬某日夜间，由被告人张某驾车并准备作案工具、被告人姜某甲指路放哨、被告人程某着手盗窃，将被害人樊某、焦某甲、焦向甲、张某甲、蒲某甲、褚某甲所有的中华田园犬各一只，被害人张某乙、郭某甲、欧某甲、焦某甲所有的狼犬各一只，被害人王某甲所有的马犬一只，被害人褚某乙所有的狮子犬一只，被害人康某甲所有的拉布拉多犬一只盗走。

2.2015 年 10 月中旬某日夜间，由被告人张某驾车、被告人姜某甲指路放哨、被告人程某着手盗窃，将害人姜某丙、卢某甲、吕某甲、冷某甲、任某甲、欧某乙、侯某甲、苟某甲、苟某乙、苟某丙所有的中华田园犬各一只，被害人王某乙所有的狮子犬、中华田园犬各一只盗走。

3.2015 年 10 月中旬某日夜间，由被告人张某驾车、被告人姜某甲指路放哨、被告人程某着手盗窃，将被害人王某丙、王某丁、张某丙所有的中华田园犬各一只盗走。

4.2015 年 10 月下旬某日夜间，由被告人姜某乙先后两次驾驶面包车并记路、被告人程某及杨姓男子着手盗窃，将被害人杨某甲、雷某甲、石某甲、张某丁所有的中华田园犬各一只，被害人李某甲、杨某乙所有的狼犬各一只，被害人曾某甲所有的哈士奇犬一只盗走。

5.2015 年 10 月下旬某日夜间，由被告人姜某乙驾驶面包车、被告人姜某甲及杨姓男子望风、被告人程某着手盗窃，将被害人付某甲、杨某戊、刘某甲、冷某乙、韩某甲、唐某甲、周某甲所有的中华田园犬各一只，被害人何某甲所有的萨摩耶犬一只，被害人鲜某甲所有的狼犬一只，被害人任某甲所有的拉布拉多犬一只盗走；作案后，被告人姜某乙再次驾驶面包车搭乘被告人程某及杨姓男子，将被害人张某戊、袁某甲、卢某乙、索某甲、杨所有的中华田园犬各一只，被害人冷某丁所有的萨摩耶犬一只，被害人黄某乙所有的中华田园犬、哈巴犬各一只、被害人索心发所有的中华田园犬两只、被害人杨乙某所有牧羊犬一只盗走。

6.2015 年 11 月 13 日夜间至 14 日凌晨，由被告人姜某乙驾驶面包车、被告人姜某甲记路望风、被告人程某着手盗窃，将被害人白某甲、张某已、杨某已、杨某庚、郭某乙所有的中华田园犬各一只，被害人廖某甲所有的哈士奇犬一只，被害人向某乙所有的宠物犬一只盗走。

7.2015 年 11 月 14 日夜间至 15 日凌晨，由被告人姜某乙驾驶面包车、被告人姜某甲记路望风、被告人程某着手盗窃，将被害人高某甲、杨某辛、杨某壬、杨某癸、杨甲某、赵某乙、赵某丙所有的中华田园犬各一只，被害人王某戊所有的拉布拉多犬一只盗走。

8.2015 年 11 月 15 日夜间至 16 日凌晨，由被告人姜某乙驾驶面包车、被告人姜某甲记路望风、被告人程某着手盗窃，将被害人马某乙、张某庚、刘某丁所有的中华田园犬各一只，被害人吴某甲所有的罗威

纳犬一只，被害人李某丙所有的中华田园犬两只，被害人黄某甲所有的金毛犬一只，被害人陈某乙所有的杜高犬一只，被害人罗某乙所有的宠物犬一只走。

9.2015 年 11 月 16 日夜间至 17 日凌晨，由被告人姜某乙驾驶面包车、被告人姜某甲记路望风、被告人程某着手盗窃，将被害人王某乙、赵某乙、薛某乙、黎某甲、邱某甲、程某乙、胡某乙、苟某戊、伍某甲、牟某甲、牟某乙、牟某丙、白某乙所有的中华田园犬各一只，被害人薛某丙、余某甲所有的牧羊犬各一只，被害人冯某乙所有的狮子犬一只盗走。三被告人在返程途中被民警现场挡获，当场查获被盗犬只 16 只，作案工具剪线钳一把。

2015 年 10 月至 2015 年 11 月 17 日期间，被告人邓某明知被告人程某出售的犬只是其盗窃所得，仍多次予以购，金额共计 8800 元，并对收购来的犬只进行了转售，致使被盗犬只无法追回。2015 年 11 月 17 日，民警将被告人邓某挡获，在其驾驶的面包车内查获被盗犬只 17 条。

**判决：**被告人程某、张某、姜某甲、姜某乙以非法占有为目的，采取秘密窃取的方法多次盗窃他人财物，其行为均已构成盗窃罪；其中被告人程某参与作案 92 次，被告人张某参与作案 27 次，被告人姜某甲参与作案 76 次，被告人姜某乙参与作案 65 次；被告人邓某明知被告人程某出售的犬只是其盗窃所得，而仍然予以收购，其行为均已构成掩饰、隐瞒犯罪所得罪。

一、被告人程某犯盗窃罪，判处有期徒刑二年，并处罚金人民币 10000 元。

二、被告人张某犯盗窃罪，判处有期徒刑一年二个月，并处罚金人民币 5000 元。

三、被告人姜某甲犯盗窃罪，判处有期徒刑十个月，并处罚金人民币 5000 元。

四、被告人姜某乙犯盗窃罪，判处有期徒刑八个月，并处罚金人民币 5000 元。

五、被告人邓某犯掩饰、隐瞒犯罪所得罪，判处拘役六个月，并处罚金人民币 10000 元。

五、作案工具断线钳一把依法予以没收；被告人张某、姜某乙、邓某某退赔款由扣押机关依法处理。
