# Supplementary material for: Protecting Companion Animals Under Chinese Criminal Law: Current Practice and Future Paths
Source: Animals (Basel). 2026 Jul 8;16(14):2119. doi: 10.3390/ani16142119 (PMC13405461; doi:10.3390/ani16142119)
Supplement: Supplementary file 1 [file animals-16-02119-s001.zip › animals-4321148-supplementary/animals-4321148-supplementary7.3/Criminal Judgment of Case 19.pdf]

案例 19 刑事判决书

案　　　　由： 侵犯财产罪/盗窃罪
妨害社会管理秩序罪/妨害司法罪/掩饰、隐瞒犯罪所得、犯罪所得收益罪

案情： 2019 年 11 月，被告人邱某、文某与被告人祁某 1 经预谋，约定由被告人祁某 1 实施盗窃，被告人邱某、文某对所盗窃的狗予以收购、销售。经鉴定，被盗狗共计价值人民币 6400 元。被告人祁某 2 明知是被告人祁某 1 盗窃所得，仍将价值人民币 6000 元的狗送到被告人邱某、文某处出售。分述如下：

1. 2019 年 11 月 10 日 0 时许，被告人祁某 1 在窃得被害人白某的土狗 1 条卖给被告人邱某、文某。经鉴定，该狗价值人民币 400 元。

2. 2019 年 11 月 12 日 19 时许，被告人祁某 1 窃得被害人张某的金毛犬 1 条，由被告人祁某 2 卖给被告人邱某、文某。经鉴定，该金毛犬价值人民币 4600 元。

3. 2019 年 11 月 12 日 20 时许，被告人祁某 1 窃得被害人刘某的金毛犬 1 条，由被告人祁某 2 卖给被告人邱某、文某，经鉴定，该金毛犬价值人民币 1400 元。

4. 2019 年 11 月 12 日 20 时许，被告人祁某 1，将被害人庄某的 1 条土狗药死，后因被人发现而未能将狗盗走。

判决： 被告人邱某、文某、祁某 1 以非法占有为目的，经事先预谋多次秘密窃取他人财物，数额较大，其行为侵犯了公私财产所有权，已构成盗窃罪，且系共同犯罪。被告人祁某 2 明知是犯罪所得，仍予以掩饰、隐瞒，其行为妨害了社会管理秩序和国家司法机关的正常活动，已构成掩饰、隐瞒犯罪所得罪。

一、被告人邱某犯盗窃罪，判处有期徒刑八个月，并处罚金人民币四千元。

二、被告人祁某 1 犯盗窃罪，判处有期徒刑七个月，并处罚金人民币四千元。

三、被告人文某犯盗窃罪，判处有期徒刑六个月，缓刑一年，并处罚金人民币四千元。

四、被告人祁某 2 犯掩饰、隐瞒犯罪所得罪，判处拘役四个月，缓刑六个月，并处罚金人民币三千元。

五、责令被告人邱某、文某、祁某 1 于本判决生效后十日内退赔被害人白某经济损失人民币四百元、退赔被害人张某经济损失人民币四千六百元、退赔被害人刘某经济损失人民币一千四百元。
