# Supplementary material for: Protecting Companion Animals Under Chinese Criminal Law: Current Practice and Future Paths
Source: Animals (Basel). 2026 Jul 8;16(14):2119. doi: 10.3390/ani16142119 (PMC13405461; doi:10.3390/ani16142119)
Supplement: Supplementary file 1 [file animals-16-02119-s001.zip › animals-4321148-supplementary/animals-4321148-supplementary7.3/Criminal Judgment of Case 2.pdf]

案例 2 刑事判决书

案　　　　由：侵犯财产罪/故意毁坏财物罪

案情：2012 年 10 月 21 日 21 时许，被告人黄某酒后召集被告人奚某 1、晏某、奚某 2、周某（另案处理）等人，驾车并持刀、铁棍找某项目部工作人员发泄。项目部工作人员见有人持刀、棍冲入项目部，便躲入办公室，被告人黄某、奚某 1、晏某、奚某 2 等人在找不到工作人员的情况下，持刀、棍打坏项目部的门窗、盆景、厨房、宠物狗及项目部工作人员停在附近的汽车后逃离现场。经鉴定，被打坏的汽车损坏修复价格共计人民币 24200 元。

判决：被告人黄某、奚某 1、晏某、奚某 2 故意非法毁坏公私财物，数额较大，其行为均已构成故意毁坏财物罪。

一、对于被告人黄某，判处有期徒刑十个月。

二、对于被告人奚某 1，判处有期徒刑八个月。

三、对于被告人晏某犯，判处有期徒刑七个月，缓刑一年。

四、对于被告人奚某 2，判处有期徒刑七个月，缓刑一年。
