# Supplementary material for: Protecting Companion Animals Under Chinese Criminal Law: Current Practice and Future Paths
Source: Animals (Basel). 2026 Jul 8;16(14):2119. doi: 10.3390/ani16142119 (PMC13405461; doi:10.3390/ani16142119)
Supplement: Supplementary file 1 [file animals-16-02119-s001.zip › animals-4321148-supplementary/animals-4321148-supplementary7.3/Criminal Judgment of Case 20.pdf]

案例 20 刑事判决书

案　　　　由：危害公共安全罪/投放危险物质罪

案情：被告人邹某因曾在某小区被狗咬伤，便用氟乙酸类鼠药浸泡鸡肝，于 2017 年 8 月 28 驾驶电动三轮车至该小区，将毒鸡肝投放在其儿子邹某某所租车库附近的草坪上，导致该小区居民周某、于某、陈某、张某、陈某饲养的六只宠物犬误食毒鸡肝死亡。经鉴定，死亡的六只宠物犬胃内容物提取物及该小区内遗留的可疑鸡肝中均含有的氟乙酸类杀鼠剂成分。

辩护意见：被告人邹某辩称，2017 年夏天，其去儿子居住的小区时被一只流浪狗咬伤，因担心家人被狗伤害，就去市场买了老鼠药和鸡肝，目的是想毒死流浪狗。其购买的老鼠药毒性也不是特别大。 辩护人辩称，被告人投放毒鸡肝的地点是小区楼栋之间的花坛树墙下，不应认定为公共场所，固体鸡肝不会对空气、土壤、水体产生毒害，且被告人并无投放危险物质危害公共安全的主观故意，本案应认定其为故意毁坏财物罪。

判决：被告人邹某仅凭自身喜恶，故意投放危险物质，危害公共安全，尚未造成严重后果，其行为已构成投放危险物质罪。关于起诉书指控张某饲养的宠物犬因误食毒鸡肝死亡的事实，因公诉机关未提供该宠物犬胃内容物检验报告，无法确定死亡原因，故本院不予认定。关于辩护人主张的其构成故意毁坏财物罪的辩护意见，经查认为，小区的绿化带属于集体业主共有，且具有开放性、流动性的特点，应当认定为公共场所，被告人在草坪中投放毒鸡肝的行为侵犯的法益不单纯指向宠物犬饲主的财产权，而是足以对不特定多数人的生命、财产安全构成侵犯，故对该辩护意见本院不予采纳。判处有期徒刑三年。
