# Supplementary material for: Protecting Companion Animals Under Chinese Criminal Law: Current Practice and Future Paths
Source: Animals (Basel). 2026 Jul 8;16(14):2119. doi: 10.3390/ani16142119 (PMC13405461; doi:10.3390/ani16142119)
Supplement: Supplementary file 1 [file animals-16-02119-s001.zip › animals-4321148-supplementary/animals-4321148-supplementary7.3/Criminal Judgment of Case 21.pdf]

案例 21 刑事判决书

案　　　　由：危害公共安全罪/投放危险物质罪

案情：被告人沙某、王某均系某小区居民，王某系该小区保安。2021 年 11 月之前，沙某、王某因不满小区业主在溜宠物犬过程中放任宠物犬在小区草坪上肆意拉尿，二人预谋向小区草坪等公共区域投放涂抹鼠药的食物毒死宠物犬。2021 年 11 月 4 日，沙某让其朋友林某购买含有氟乙酸类成分的鼠药后，沙某将购买的 2 瓶鼠药交给王某，王某购买鸡肝并用鼠药浸泡。同月 6 日 18 时许，王某利用其值班之时，将浸泡鼠药的鸡肝投放至该小区东侧及Ａ6 号楼与Ａ3 号楼之间草坪上，导致 11 条宠物犬中毒死亡。经牡丹江市刑事技术支队检验：送检疑似鼠药液体中检出氟乙酸根离子；涉案 11 条宠物犬的胃内容及心脏血中均检出氟乙酸类鼠药成分；疑似动物肝脏中检出氟乙酸类鼠药成分；王某上衣衣袖中检出氟乙酸类鼠药成分。经价格认证中心认定：涉案 11 条宠物犬（拉布拉多犬、雌性边境牧羊犬、吉娃娃犬、咖啡白色泰迪犬、灰色泰迪犬、黄色泰迪犬、黑色泰迪犬、八哥犬、柯基犬、雄性边境牧羊犬在 2021 年 11 月 6 日的市场价格共计 34300 元；黄色泰迪犬在 2021 年 11 月 15 日的市场价格为 1000 元。

辩护意见：

被告人沙某辩护人提出的辩护意见：1.沙某已自愿认罪认罚，可对其从宽处理；2.本案的犯意促成者不是沙某，其没有危害公共安全的主观故意，主观恶性较小；3.沙某系初犯，没有严重的前科劣迹，已与本案被害人达成和解并取得被害人的谅解，其人品好，与小区内的邻居相处和谐，积极参与疫情防控，小区多名业主联名书写材料请求法院对沙某从轻或减轻处罚；4.本案被害人存在过错，不按照饲养家犬的相关规定对家犬进行圈养、拴绳管理，在小区内散放，在草坪里和人行道路上肆意拉尿，造成小区环境卫生脏乱差，而引发了二被告人犯罪；5.本案证据没有达到刑事案件证据确实、充分的标准，缺少物证即装鼠药的瓶子，不能证明二被告人用于毒害家犬的药水一定是具有巨毒的氟乙酸类鼠药，不能排除是他人抛散了拌有氟乙酸类鼠药成分鸡肝的合理怀疑；6.对沙某的量刑重于王某不妥，沙某通过林某 1 将不明成分的鼠药捎来后交给王某，王某实施了买鸡肝拌鼠药投放到草坪的行为，造成 11 条狗死亡的结果。

被告人王辩护人提出的辩护意见：1.公诉机关起诉罪名错误，在案证据不能证明王某构成投放危险物质罪，应认定为故意毁坏财物罪，量刑建议畸重；2.公诉机关指控遗漏他人指使犯罪的事实，应认定王某是从犯；3.王某到案后如实供述犯罪事实及同案犯，可对其从轻处罚；4.王某已全部赔偿被害人的财产损失并取得被害人谅解，可对其酌情从轻处罚；5.王某认罪认罚，可对其从宽处理。

被告人王某的辩护人举示证据：2021 年 11 月 6 日天气情况截图打印件，证明当天是雨夹雪的恶劣天气，拌有耗子药的鸡肝被掰成指甲盖大小后扔到草坪上会和泥水混为一体，同时会被积雪覆盖，结合本案证据，不存在人会捡食的情况，也不存在危害公共安全。尤其是投放鼠药的草地，属于限制人员随意进入的公共区域，在此地投放浸有鼠药的鸡肝不应认定为危害公共安全。公诉机关对此份证据的形式要件没有异议，对证明的问题有异议，认为案发当天是雨夹雪天气，并不影响鼠药的毒性，而且从整个案件后果来看，涉案 11 条狗已经中毒身亡，说明对公共安全的危害存在。

判决：被告人沙某、王某向公共区域投放毒害性物质，危害公共安全，尚未造成严重后果，二被告人的行为已构成投放危险物质罪。

关于被告人沙某的辩护人提出本案的犯意者不是沙某，其没有危害公共安全的主观故意，主观恶性较小及被告人王某的辩护人提出在案证据不能证明王某构成投放危险物质罪，公诉机关起诉罪名错误的辩护意见，投放危险物质行为的对象是不特定的公私财物，包括房屋、牲畜、家禽、机动车辆等财物，而故意毁坏财物行为指向一般是特定的财物，投放危险物质行为的危害后果是危害了公共安全，而故意毁坏财物行为的危害后果一般是造成了特定的财物毁损。沙某、王某将浸有鼠药的鸡肝扔在小区草坪的公共区域，其想要危害的不是特定的某只犬或其他财物、动物，只要从该小区草坪经过的动物都有可能误食并可能造成死亡的后果，还有可能在动物身体上沾有鼠药并传染到动物主人的身上并造成伤害，二被告人的主观目的是想要毒死小区内随意拉尿的犬类，但其采用的手段客观上造成了危害公共安全的后果，故沙某的辩护人及王某的辩护人提出的此点辩护意见不成立，本院不予采纳；关于沙某、王某自愿认罪认罚，可对其从宽处理的辩护意见符合法律规定，本院予以采纳；关于沙某、王某已与本案全部被害人达成和解，并取得被害人谅解，可酌情从轻处罚的辩护意见，符合法律规定，本院予以采纳；关于沙某的辩护人提出沙某系初犯，没有严重的前科劣迹，且其与小区内的邻居相处和谐，积极参与疫情防控，小区多名业主联名书写材料请求法院对沙某酌情从轻处罚的辩护意见，本院予以采纳；关于沙某的辩护人提出本案被害人存在过错，不按照饲养家犬的相关规定对家犬进行圈养、拴绳管理，而引发被告人犯罪的辩护意见，不是沙某可以触犯法律犯罪的理由，本院不予采纳；关于本案缺少物证即装鼠药的瓶子，不能证明二被告人用于毒害家犬的药水一定是具有巨毒的氟乙酸类鼠药，不能排除是他人抛撒了拌有氟乙酸类鼠药成分鸡肝的合理怀疑的辩护意见，本案在案证据形成了完整的证据链条，并与二被告人的当庭供述一致，故此点辩护意见不成立，本院不予采纳；关于沙某的辩护人提出对沙某量刑重于王某不妥的辩护意见，因本案系共同犯罪，沙某负责购买鼠药，王某负责买鸡肝拌鼠药投放到草坪，二被告人各有分工，在共同犯罪中均系主犯，且沙某在侦查阶段及检察阶段均未认罪认罚，综合考量二被告人的犯罪情节、认罪态度、悔罪表现等，将被告人沙某列到前位并无不妥，此点辩护意见不成立，本院不予采纳；王某的辩护人关于公诉机关指控本案遗漏他人指使犯罪的事实，应认定王某系从犯的辩护意见，与本案查明的事实不符，且无其他证据予以证实，本院不予采纳；关于王某到案后如实供述犯罪事实及同案犯，可对其从轻处罚的辩护意见符合法律规定，本院予以采纳。

一、对于被告人沙某，判处有期徒刑三年七个月；

二、对于被告人王某，判处有期徒刑三年六个月。
