# Supplementary material for: Protecting Companion Animals Under Chinese Criminal Law: Current Practice and Future Paths
Source: Animals (Basel). 2026 Jul 8;16(14):2119. doi: 10.3390/ani16142119 (PMC13405461; doi:10.3390/ani16142119)
Supplement: Supplementary file 1 [file animals-16-02119-s001.zip › animals-4321148-supplementary/animals-4321148-supplementary7.3/Criminal Judgment of Case 22.pdf]

案例 22 刑事判决书

案　　　由：危害公共安全罪/投放危险物质罪

案情：被告人韩某在某停车场跑步时踩到宠物狗的粪便，对宠物狗产生报复心理，于 2020 年 2 月 23 日下午，购买一包毒鼠强和一块熟鸡肝，将鸡肝弄碎掺入毒鼠强装入塑料袋中。当日 17 时许，韩某到该停车场，将掺有毒鼠强的鸡肝撒在停车场入口石球附近，后离开停车场。当日 17 时许，刘某等七人先后到该停车场附近遛狗，回家后，七条宠物狗出现中毒症状先后死亡。刘某到该停车场石球附近拾到了鸡肝，经检验：部分鸡肝中检出氟乙酸成分。案发后，公安机关提取了宠物狗的内脏，经检验：所送部分狗内脏组织 1#和 2#中均检出氟乙酸成份。

辩护意见：被告人的行为不构成投放危险物质罪，其主观上是故意毁坏财物；被告人具有自首情节，可从轻处罚。

判决：被告人韩某在公共场所投放毒害性物质，危害公共安全，尚未造成严重后果，其行为已构成投放危险物质罪。辩护人关于被告人主观上仅具有故意毁坏他人财物的故意、其行为不构成投放危险物质罪的辩解意见，经查，停车场附近是市民（大人、小孩）及宠物经常活动的地方，被告人韩某为泄私愤，故意将含有毒鼠强的熟鸡肝投放在该公共场所，当日即造成七条大型宠物狗死亡的后果，更威胁了不特定人、畜的生命、财产安全，其行为已构成投放危险物质罪，辩护人的辩解意见，不予采纳。辩护人关于被告人构成自首的辩解意见，于法有据，予以采纳。判处有期徒刑三年。
