# Supplementary material for: Protecting Companion Animals Under Chinese Criminal Law: Current Practice and Future Paths
Source: Animals (Basel). 2026 Jul 8;16(14):2119. doi: 10.3390/ani16142119 (PMC13405461; doi:10.3390/ani16142119)
Supplement: Supplementary file 1 [file animals-16-02119-s001.zip › animals-4321148-supplementary/animals-4321148-supplementary7.3/Criminal Judgment of Case 23.pdf]

案例23 刑事判决书

案　　　　由：危害公共安全罪/投放危险物质罪

案情：2018年7月27日22时许，被告人刘某在某住宅区，将掺有鼠药的熟鸡肝块扔在该小区自己别墅院内及刘某家门前楼梯附近。当晚及次日，该小区居民邵某、闫某、贾某、邓某、冉某、张某、陈某、孟某家的宠物犬在食用上述鸡肝块后相继死亡。经价格认证中心认证，死亡的八只宠物犬共计损失价值人民币8200元，其中闫某家的泰迪犬价值1100元、贾某家的泰迪犬价值人民币900元、邵某家的泰迪犬价值1300元、邓某家的鹿娃犬价值人民币1500元、张某家的京巴犬价值人民币500元、冉某家的金毛犬价值人民币900元、陈某家的比熊犬价值人民币1000元、孟某家的泰迪犬价值人民币1000元。

附带民事诉讼情况：

被害人暨附带民事诉讼原告人贾某请求依法追究被告人刘某的刑事责任，同时请求判令被告人刘某赔偿经济损失人民币15000元。

被害人暨附带民事诉讼原告人张某请求依法追究被告人刘某的刑事责任，同时请求判令被告人刘某赔偿经济损失人民币10000元。

被害人暨附带民事诉讼原告人邵某请求依法追究被告人刘某的刑事责任，同时请求判令被告人刘某赔偿经济损失人民币7983元。

被害人暨附带民事诉讼原告人冉某请求依法追究被告人刘某的刑事责任，同时请求判令被告人刘某赔偿经济损失人民币3000元。

被害人暨附带民事诉讼原告人孟某请求依法追究被告人刘某的刑事责任，同时请求判令被告人刘某赔偿经济损失人民币5000元。

辩护意见：被告人刘某辩称，投放鼠药的原因是他的孙女之前被小区内的狗扑倒，吓着了，没有找到狗的主人，以为是流浪狗；投放鼠药的位置是流浪狗过夜的地方，没有向公共场所和绿化带内投放鼠药，不构成投放危险物质罪；对各附带民事诉讼原告人主张的经济损失，不同意赔偿。辩护人认为，被告人刘某投放鸡肝的地点不具有公共性；投放的行为不具有危害公共安全的危险性；对于毒死8条狗，主观没有故意，公诉机关出示的证据不能证明被告人刘某构成投放危险物质罪。

判决：被告人刘某故意投放危险物质，危害公共安全，尚未造成严重后果，其行为已构成投放危险物质罪。被告人刘某案发后主动投案，能够如实供述自己的罪行，庭审中其虽辩解不构成投放危险物质罪，是其认识理解错误，应认定为自首，可以从轻处罚；案发后主动赔偿部分被害人并取得谅解，可酌情从轻处罚。被告人及其辩护人提出被告人不构成投放危险物质罪的相关辩解或辩护意见，与事实与法律规定不符，不能成立，不予采纳。辩护人提出被告人具有自首情节的辩护意见成立，予以采纳。被告人刘某不同意赔偿被害人损失的辩论意见，不符合事实与法律规定，不予采纳。

一、对于被告人刘某，判处有期徒刑三年六个月。

二、被告人刘某赔偿各附带民事诉讼原告人经济损失共计人民币4600元，其中贾某人民币900元、张某人民币500元、邵某人民币1300元、冉某人民币900元，孟某人民币1000元，于本判决发生法律效力后立即给付。

三、驳回附带民事诉讼原告人贾某、张某、邵某、冉某、孟某其他诉讼请求。
