# Supplementary material for: Protecting Companion Animals Under Chinese Criminal Law: Current Practice and Future Paths
Source: Animals (Basel). 2026 Jul 8;16(14):2119. doi: 10.3390/ani16142119 (PMC13405461; doi:10.3390/ani16142119)
Supplement: Supplementary file 1 [file animals-16-02119-s001.zip › animals-4321148-supplementary/animals-4321148-supplementary7.3/Criminal Judgment of Case 24.pdf]

案例 24 刑事裁定书

案　　　　由：危害公共安全罪/投放危险物质罪

案情：被告人彭某原系某小区物业保洁员。因该小区业主遛狗时不清理狗粪问题，被告人曾与业主多次发生矛盾，后遂产生毒狗报复心理。2019 年 5 月 12 日、5 月 13 日，被告人彭某在该小区 C 座楼下绿化带里，投放了十几块用“老鼠药”浸泡过的绿色熟肉。后公安机关在彭某临时住处搜查出绿色液体一瓶，在案发现场绿化带里提取到绿色熟肉五块。经鉴定，在绿色液体和熟肉中检出氟乙酸根离子成分。

一审判决：被告人彭某未能妥善处理与他人矛盾，为泄私愤故意在小区公共绿地投放有毒物质，危害公共安全，尚未造成严重后果，已构成投放危险物质罪。关于辩护人提出的被告人不构成投放危险物质罪的意见，原审法院认为小区的绿化带属于集体业主共有，且具有开放性、流动性的特点，应当认定为公共场所，被告人在草坪中投放用“老鼠药”浸泡过的绿色熟肉的行为侵犯的法益不单纯指向与其发生矛盾的宠物犬饲主的财产权，而是足以对不特定多数人的生命、财产安全构成侵犯，故对该辩护意见本院不予采纳。被告人彭某犯投放危险物质罪，判处有期徒刑三年。

上诉理由：其投放的有毒物质主要针对特定业主的宠物狗，不易对其他人造成伤害。其系自首，认罪态度较好，其行为亦未造成人员损害，原审量刑过重，请求二审法院减轻处罚。

辩护意见：从方法和投放的位置来看，上诉人彭某投放的危险物质并不危害公共安全，其主观目也不是为了伤害不特定其他的人身和财产安全，其本身对后果持过失心态，请求二审法院考虑彭某具备自首，认罪认罚等情节，请求从轻处罚。

二审裁定：上诉人彭某故意在居住社区公共绿地上投放毒害性物质，危害公共安全，其行为构成投放危险物质罪。原审法院认定上诉人彭某构成自首并予以从轻处罚，均无不当。 关于上诉人彭某提出其投放的危险物质针对特定业主的宠物狗，不易对其他人造成伤害以及其辩护人提出的其行为不足以危及公共安全的辩护意见，经查，小区的绿化带作为公共场所，具备开放性和公共属性的特征，上诉人彭某投放的具体位置看，有毒物质颗粒较小，没有与外界完全隔绝，尚不能完全避免接触和误食风险。上诉人投放危险物质虽有一定程度上的指向性，但其主观上对可能造成其他损害结果持放任心态，且损害的范围和实际的后果是其难以控制的，可能导致不特定多数人的生命健康或重大财产遭受危险，属于侵害公共安全的行为，故上诉人及其辩护人认为其行为没有危及公共安全的上诉理由和辩护意见没有事实和法律依据，本院不予采纳。关于上诉人彭某及其辩护人提出的系自首，主观恶性较小的上诉理由和辩护意见，经查，原审已经认定彭某构成自首并综合全案情况予以从轻处罚，所处刑罚适当，故该上诉理由和辩护意见本院不予采纳。综上，原判认定事实清楚，证据确实充分，定罪准确，量刑适当，审判程序合法；驳回上诉，维持原判。
