# Supplementary material for: Protecting Companion Animals Under Chinese Criminal Law: Current Practice and Future Paths
Source: Animals (Basel). 2026 Jul 8;16(14):2119. doi: 10.3390/ani16142119 (PMC13405461; doi:10.3390/ani16142119)
Supplement: Supplementary file 1 [file animals-16-02119-s001.zip › animals-4321148-supplementary/animals-4321148-supplementary7.3/Criminal Judgment of Case 25.pdf]

案例 25 刑事判决书

案　　　　由：危害公共安全罪/危险驾驶罪

案情：2018 年 6 月 20 日许，被告人李某醉酒后驾驶轻型普通货车，与被害人李某未牵绳的宠物狗相碰撞，致狗死亡。经鉴定，被告人李某静脉血中乙醇成分含量为 93.29mg/100ml。案发后，被告人赔偿被害人损失 3000 元。

判决：被告人李某在道路上醉酒驾驶机动车，其行为已构成危险驾驶罪；判处拘役一个月，并处罚金人民币二千元。
