# Supplementary material for: Protecting Companion Animals Under Chinese Criminal Law: Current Practice and Future Paths
Source: Animals (Basel). 2026 Jul 8;16(14):2119. doi: 10.3390/ani16142119 (PMC13405461; doi:10.3390/ani16142119)
Supplement: Supplementary file 1 [file animals-16-02119-s001.zip › animals-4321148-supplementary/animals-4321148-supplementary7.3/Criminal Judgment of Case 26.pdf]

案例 26 刑事判决书

案　　　　由：危害公共安全罪/交通肇事罪

案情：2020 年 5 月 6 日 12 时 29 分许，被告人陈某饮酒后驾驶轿车，自东向西超速（限速 40km／h，实速 51km／h-54km／h）行经某地段，碰撞牵引宠物犬自北向南横过机动车道的行人林某，造成林某受伤经抢救无效于次日死亡、宠物犬死亡及车辆损坏的道路交通事故。事故发生后，被告人陈某为掩盖其饮酒后驾驶机动车的事实而弃车逃逸。经事故责任认定，被告人陈某承担事故的主要责任，林某一承担事故的次要责任。

判决：被告人陈某违反交通运输管理法规，因而发生重大事故，致一人死亡，负事故的主要责任，且肇事后逃逸，其行为已构成交通肇事罪；判处有期徒刑三年六个月。
