# Supplementary material for: Protecting Companion Animals Under Chinese Criminal Law: Current Practice and Future Paths
Source: Animals (Basel). 2026 Jul 8;16(14):2119. doi: 10.3390/ani16142119 (PMC13405461; doi:10.3390/ani16142119)
Supplement: Supplementary file 1 [file animals-16-02119-s001.zip › animals-4321148-supplementary/animals-4321148-supplementary7.3/Criminal Judgment of Case 27.pdf]

案例 27 刑事判决书

案　　　　由：危害公共安全罪/非法持有、私藏枪支、弹药罪

案情：2013 年 5 月 31 日 17 时许，被告人吴某在居住小区内，由于自家宠物猫遭到邻居的大型犬攻击，遂持一把私藏的枪支将狗打伤。当晚 22 时许，被告人吴某伙同林某（另案处理）将其非法持有的二把枪支、子弹转移至其租住的其他住房进行藏匿，后民警将二人抓获归案，并缴获上述枪支 2 把、弹药 510 发。经鉴定，缴获的枪支一把为以火药为动力发射弹丸的制式唧筒式猎枪，另一把为以火药为动力发射弹丸的制式 5.6 毫米口径运动步枪；缴获的 510 发子弹中有 310 发为制式 12 号猎枪弹，200 发为制式 5.6 毫米小口径枪弹。

判决：被告人吴某违反枪支管理规定，非法持有枪支、弹药，情节严重，其行为已构成非法持有枪支、弹药罪；判处有期徒刑三年，缓刑五年；缴获的枪支 2 把、弹药 510 发，予以没收，依法销毁。
