# Supplementary material for: Protecting Companion Animals Under Chinese Criminal Law: Current Practice and Future Paths
Source: Animals (Basel). 2026 Jul 8;16(14):2119. doi: 10.3390/ani16142119 (PMC13405461; doi:10.3390/ani16142119)
Supplement: Supplementary file 1 [file animals-16-02119-s001.zip › animals-4321148-supplementary/animals-4321148-supplementary7.3/Criminal Judgment of Case 28.pdf]

案例 28 刑事判决书

案　　　　由：破坏社会主义市场经济秩序罪/生产、销售伪劣商品罪/生产、销售有毒、有害食品罪

案情：被告人代某与其妻子蒲某 1 长期从事食品的收购和销售经营。2015 年 3 月 22 日下午，被告人代某、蒲某 1 夫妇在收购死狗时被公安机关查获，8 条死狗、11 袋冷冻的狗肉(共重 79.85 千克)亦被查获。经鉴定，被查获的冷冻狗肉中检出有毒物质琥珀胆碱。2015 年 3 月 20 日、3 月 21 日，被告人蒲某 2 以毒针射杀的方式盗得土狗 4 条，以 4 元/斤的价格销售给代某、蒲某 1，获利 300 余元。2015 年 3 月 22 日，被告人蒲某 2 伙同杨某以毒箭射杀的方式盗得 8 条狗（共重 160 余斤），后以 4 元/斤的价格销售给代某、蒲某 1 时，被公安机关人赃俱获。公安机关当场从被告人蒲某 2 处查获盗狗工具机械弓弩 1 把，箭 6 支。经鉴定，箭中的水剂均含有氯化琥珀胆碱。2015 年春节前后，被告人蒋某通过“三步倒”盗得土狗 20 余条，再以 4 元/斤的价格销售给代某、蒲某 1。

辩护意见：

被告人代某、蒲某 1 辩称：不知道收购和销售的死狗里边有毒。

辩护人的辩护意见：公诉机关指控的罪名不能成立，应当构成销售不符合安全标准的食品罪。死狗有些是有毒的，有些是没有的，从被告人的生活知识无法知道死狗是有毒的。公诉机关指控的销售数量是推理得来的，也是不合理。

被告人蒲某 2 辩称：不知道射死的狗有毒，后来鉴定后才知道的。

被告人杨某辩称：只是跟着蒲某 2 去了一次，不知道死狗有毒。

被告人蒋某辩称：不知道死狗有毒，“三步倒”弄死的狗只是晕倒，自己也吃了这种狗的。

辩护人的辩护意见：“三步倒”是否是有毒物质公诉机关没有提供相印的证据证实；“三步倒”弄死的狗是否有毒也无证据证实；“三步倒”弄死的狗被告人和证人都吃过，没有发现问题。被告人蒋某自己供述了用“三步倒”弄死了狗，但没有查获到死狗，也没有查获“三步倒”，没有相关证据与被告人蒋某的供述相印证，只有被告人的供述。从本案的事实看，本案的事实不清，证据不足，依照疑罪从无的原则，应当判决被告人蒋某无罪。

判决：被告人代某、蒲某 1、蒲某 2、杨某、蒋某应当明知是掺有有毒、有害非食品原料的食品（死狗），仍然予以销售，严重危害了公共食品安全、破坏了市场经济秩序，应当以销售有毒、有害食品罪追究其刑事责任。被告人代某、蒲某 1 已经销售的狗肉，公诉机关没有提供充分证据证明其中含有毒、有害非食品原料，对于该部分，本院不予认定为公诉机关指控的“掺有有毒、有害非食品原料的食品”的指控事实。对于查获被告人代某、蒲某 1 未销售的含有琥珀胆碱的狗肉，应当认定为销售有毒、有害食品的未遂行为。因为被告人代某、蒲某 1 有销售有毒、有害食品的故意，也在组织实施销售活动，其食品（死狗）中也检测出了琥珀胆碱的非食品原料，由于被公安机关当场查获，属于意志以处的原因而未能得逞，是犯罪未遂，可以比照既遂犯从轻或减轻处罚。辩护人辩称“公诉机关指控的罪名不能成立，应当构成销售不符合安全标准的食品罪”的辩解理由不能成立，不予以采纳。被告人杨某与被告人蒲某 2 用含有琥珀胆碱的药剂射杀并销售死狗 8 只，其行为构成了销售有毒、有害食品罪，但被告人杨某的犯罪情节轻微，

可以免予刑事处罚。被告人杨某辩称自己不构成销售有毒、有害食品罪的辩解意见不能成立，本院不予以采纳。被告人蒋某所用的“三步倒”，公诉机关没有提供充分证据证实是否是有毒、有害的非食品原料；被告人蒋某、蒲陶均供述对“三步倒”弄死的狗进行过食用。但从被告人蒋某的认知能力应当知道用“三步倒”弄死的狗可能含有有毒、有害成分，所以被告人蒋某的行为亦构成销售有毒、有害食品罪。但被告人蒋某的犯罪情节轻微，可以免予刑事处罚。辩护人认为被告人不构成销售有毒、有害食品罪的辩解意见不成立，不予以采纳。

一、被告人代某犯销售有毒品、有害食品罪（未遂），判处有期徒刑一年，缓刑一年零六个月，并处罚金 10000 元。

二、被告人蒲某 1 犯销售有毒品、有害食品罪（未遂），判处有期徒刑一年，缓刑一年零六个月，并处罚金 10000 元。

三、被告人蒲某 2 犯销售有毒品、有害食品罪，判处有期徒刑六个月，缓刑一年，并处罚金 5000 元。

四、被告人杨某犯销售有毒品、有害食品罪，免予刑事处罚。

五、被告人蒋某犯销售有毒品、有害食品罪，免予刑事处罚。

六、对扣押被告人代某、蒲某 1 的死狗 8 只、11 袋冻狗肉（79.85 千克）予以没收；对扣押被告人蒲某 2 的机械弓弩一把、箭六支予以没收，对被告人蒲某 2 的非法所得 300 元予以追缴。

七、被告人代某、蒲某 1、蒲某 2 在缓刑考验期内不得从事食品生产、销售及相关活动。
