# Supplementary material for: Protecting Companion Animals Under Chinese Criminal Law: Current Practice and Future Paths
Source: Animals (Basel). 2026 Jul 8;16(14):2119. doi: 10.3390/ani16142119 (PMC13405461; doi:10.3390/ani16142119)
Supplement: Supplementary file 1 [file animals-16-02119-s001.zip › animals-4321148-supplementary/animals-4321148-supplementary7.3/Criminal Judgment of Case 29.pdf]

# 案例 29 刑事判决书

**案　　　　由：**破坏社会主义市场经济秩序罪/生产、销售伪劣商品罪/生产、销售伪劣产品罪
破坏社会主义市场经济秩序罪/侵犯知识产权罪/假冒注册商标罪

**案情：**2020 年 2 月起，被告人纪某、孙某为牟取非法利益，在不具备生产资质、未经注册商标所有人许可的情况下，或单独或结伙生产、销售假冒、不合格兽药。其中，被告人纪某委托刘某 1、刘某 2、张某（均另案处理）等人生产假冒“Nobivac”商标猫三联疫苗、猫干扰素 ω、猫瘟康、呼舒坦注射液、马克西金 0.15、假冒“妙三多”商标猫用疫苗等兽药，从康某、杨某（均另案处理）等人处购入产品包装，由孙某负责联系购买假冒“Nobivac”商标的猫三联疫苗标签，最后由纪某、孙某分别安排刘某 3、彭某（均另案处理）等人贴标，并分别通过微信对外销售。此外，被告人孙某在不具备生产资质的情况下，指使员工生产 Vibravet 抗病菌消炎膏、派克消炎膏、派克眼膏、素高捷眼膏等兽药，并通过微信对外销售。2021 年 4 月 8 日，侦查机关扣押纪某销售给徐某（另案处理）的猫三联疫苗；在被告人纪某的加工点、办公点扣押假冒“Nobivac”商标猫三联疫苗、呼舒坦注射液、假冒“妙三多”商标猫用疫苗、假冒“妙三多”商标标签等物品；在刘某的车库内扣押猫干扰素 ω；在张某的厂房内扣押呼舒坦注射液、马克西金 0.15 等兽药；在被告人孙某的加工点、经营点处扣押 Vibravet 抗病菌消炎膏、派克消炎膏、派克眼膏等兽药。经检测，从扣押的生物制品猫三联疫苗中检出化学药品氨苄西林，从扣押的呼舒坦注射液、猫干扰素 ω、派克消炎膏等兽药中检出的成分与标称成分不符。经认定，上述兽药均为假兽药。经统计，被告人纪某处的不合格兽药的货值金额共计人民币 137,665.16 元（以下币种均为人民币），已销售金额共计 1,603,747.00 元，假冒注册商标的商品的非法经营数额共计 11,969,106.50 元；被告人孙某处的不合格兽药的货值金额共计 266,926.33 元，已销售金额共计 1,071,253.33 元，假冒注册商标的商品的非法经营数额共计 11,557,806.50 元。

**判决：**被告人纪某、孙某为牟取非法利益，决定并指使他人将不合格的兽药冒充合格产品进行生产、销售，其中销售金额分别为 160 万余元、107 万余元，尚未销售的货值金额分别为 13 万余元、26 万余元，其行为均已构成生产、销售伪劣产品罪；被告人纪某、孙某未经注册商标所有人的许可，在同一种商品上使用与其注册商标相同的商标并予以销售，非法经营数额分别为 1,196 万余元、1,155 万余元，情节特别严重，其行为均已构成假冒注册商标罪。被告人纪某、孙某在判决宣告前一人犯二罪，均应当实行数罪并罚。被告人已经着手实行犯罪，由于意志以外的原因部分伪劣兽药尚未销售，系犯罪未遂，可以比照既遂犯从轻或者减轻处罚。

一、被告人纪某犯生产、销售伪劣产品罪，判处有期徒刑七年三个月，并处罚金人民币九十万元；犯假冒注册商标罪，判处有期徒刑五年，并处罚金人民币三百一十万元，决定执行有期徒刑八年六个月，并处罚金人民币四百万元。

二、被告人孙某犯生产、销售伪劣产品罪，判处有期徒刑五年六个月，并处罚金人民币七十万元；犯假冒注册商标罪，判处有期徒刑四年，并处罚金人民币二百九十万元。决定执行有期徒刑七年，并处罚金人民币三百六十万元。

三、违法所得予以追缴，扣押在案的涉案假兽药及供犯罪所用的本人财物等予以没收。
