# Supplementary material for: Protecting Companion Animals Under Chinese Criminal Law: Current Practice and Future Paths
Source: Animals (Basel). 2026 Jul 8;16(14):2119. doi: 10.3390/ani16142119 (PMC13405461; doi:10.3390/ani16142119)
Supplement: Supplementary file 1 [file animals-16-02119-s001.zip › animals-4321148-supplementary/animals-4321148-supplementary7.3/Criminal Judgment of Case 3.pdf]

案例 3 刑事判决书

案　　　　由：侵犯财产罪/故意毁坏财物罪

案情：2015 年 9 月 10 日 23 时许，被告人孙某在被害人李某住处，与被害人李某发生争执，后被告人孙某殴打被害人李某，将被害人李某住处的电视机及其手机砸坏，将被害人李某饲养的一只宠物犬摔死。经鉴定，被毁坏的电视机损失价格为 2295 元；被毁坏的手机损失价格为 3800 元。

判决：被告人孙某故意毁坏他人财物，数额较大，其行为已构成故意毁坏财物罪；判处有期徒刑六个月。
