# Supplementary material for: Protecting Companion Animals Under Chinese Criminal Law: Current Practice and Future Paths
Source: Animals (Basel). 2026 Jul 8;16(14):2119. doi: 10.3390/ani16142119 (PMC13405461; doi:10.3390/ani16142119)
Supplement: Supplementary file 1 [file animals-16-02119-s001.zip › animals-4321148-supplementary/animals-4321148-supplementary7.3/Criminal Judgment of Case 30.pdf]

# 案例 30 刑事判决书

**案　　　　　由**：危害公共安全罪/非法携带枪支、弹药、管制刀具、危险物品危及公共安全罪
妨害社会管理秩序罪/扰乱公共秩序罪/寻衅滋事罪

**案情**：2013 年 12 月某日 16 时许，被告人桂某为达到强行租用农村土地的目的，携带礼花弹等爆炸物至某村村委会，采用持爆炸物并扬言若不达目的就引爆的方式，言语恐吓该村村长乔某，后被他人劝离。2013 年 12 月 20 日 10 时许，被告人桂某为达到强行排挤租用农村土地竞争对手的目的，使用言语威胁及用匕首刺伤宠物的方式恐吓被害人孙某。2014 年 3 月 14 日 20 时许，被告人桂某为达到上述相同目的，驾车截停被害人朱乙，并用毛巾包裹三脚架假冒枪支，对被害人进行恐吓威胁。2014 年 4 月 23 日 23 时 30 分许，被告人桂某酒后为逞强好胜，手持用毛巾包裹的三脚架假冒枪支恐吓被害人何兵等人，遭店内员工劝阻后，又持雷管等爆炸物恐吓店内员工谭小平等人。

**判决**：被告人桂某非法携带爆炸性物品进入公共场所，危及公共安全，情节严重，因妨害公务被刑事处罚后，又多次实施恐吓他人的行为，情节恶劣，其行为分别构成非法携带危险物品危及公共安全罪和寻衅滋事罪，依法予以两罪并罚。被告人桂某犯非法携带危险物品危及公共安全罪，判处有期徒刑六个月；犯寻衅滋事罪，判处有期徒刑一年，决定执行有期徒刑一年二个月。
