# Supplementary material for: Protecting Companion Animals Under Chinese Criminal Law: Current Practice and Future Paths
Source: Animals (Basel). 2026 Jul 8;16(14):2119. doi: 10.3390/ani16142119 (PMC13405461; doi:10.3390/ani16142119)
Supplement: Supplementary file 1 [file animals-16-02119-s001.zip › animals-4321148-supplementary/animals-4321148-supplementary7.3/Criminal Judgment of Case 31.pdf]

案例31刑事判决书

案　　　　由：侵犯公民人身权利、民主权利罪/故意伤害罪
妨害社会管理秩序罪/扰乱公共秩序罪/寻衅滋事罪

案情：2013年6月至8月间，被告人李某多次伙同马某、贝某、郭某、李某2、袁某、李某1在房屋征收过程中，多次毁坏被征收户财物，并于2013年8月30日持刀造成被害人初某1重伤。其具体犯罪事实如下：

（一）故意伤害的事实

2013年8月30日11时许，被告人李某带领马某、李某1等人（均另案处理）持刀、棒等工具至某施工现场，公然持械对现场人员初某1、张某1、于某进行殴打，并打砸现场停放车辆。造成初某1二处轻微伤、一处轻伤、一处重伤；张某1一处轻微伤、一处轻伤；于某轻微伤。其中被害人初某1左胸部重伤系被告人李某持刀造成。另造成车辆损坏，被砸车辆修车费用122628元。

（二）寻衅滋事的事实

1、2013年6月7日0时许，为迫使拆迁户搬迁以达到拆迁目的，被告人李某指使马某、贝某、郭某、李某2、袁某、李某1（均另案处理）向拆迁户胡某1、黄某家院内投掷毒物，将胡某1家两条狗、黄某家一条狗毒死，价值3500元。

2、2013年6月8日0时许，被告人李某指使马某、贝某、郭某、李某2、袁某、李某1向拆迁户裴某、李某3家院内投掷毒物，将裴某家六条狗、李某3家一条狗毒死，价值2280元。

3、2013年6月11日0时许，被告人李某指使马某、贝某、郭某、李某2、袁某、李某1乘拆迁户胡某1全家人熟睡之机，驾驶铲车将胡某1家大门、杖子、仓房推毁，价值3930元。

4、2013年6月中旬一天0时许，被告人李某指使袁某、高某将拆迁户胡某1家玻璃砸碎、电扇砸坏，被毁财物价值210元。

5、2013年6月24日0时许，被告人李某指使马某、贝某、郭某、李某2、袁某乘拆迁户胡某1全家人熟睡之机，将胡某1家门前玉米地一千颗玉米苗毁坏，被毁玉米苗价值800元。

6、2013年7月份一天0时许，被告人李某指使贝某、高某将拆迁户田某家五块玻璃砸碎。

判决：被告人李某持械聚众斗殴，致一人重伤之行为，已构成故意伤害罪；李某指使他人任意损毁公私财物，情节严重之行为，已构成寻衅滋事罪。李某经常纠集他人在一起，以暴力手段，在征地、拆迁过程中多次实施违法犯罪活动，为非作恶，欺压百姓，扰乱经济、社会生活秩序，造成较为恶劣的社会影响，应当认定为“恶势力”，依法从严惩处。李某纠集他人聚众斗殴及任意损毁他人财物，且受害人初某1的重伤是由李某持刀造成，其在共同犯罪中起主要作用，系主犯，应按照其所参与以及组织、指挥的全部犯罪处罚。

一、被告人李某犯故意伤害罪，判处有期徒刑六年；犯寻衅滋事罪，判处有期徒刑六年，并处罚金人民币二万元，决定执行有期徒刑十年，并处罚金人民币二万元。

二、责令被告人李某退赔被害人胡某 1、黄某人民币 3500 元，退赔被害人裴某、李某 3 人民币 2280 元，退赔胡某 1 人民币 4940 元。
