# Supplementary material for: Protecting Companion Animals Under Chinese Criminal Law: Current Practice and Future Paths
Source: Animals (Basel). 2026 Jul 8;16(14):2119. doi: 10.3390/ani16142119 (PMC13405461; doi:10.3390/ani16142119)
Supplement: Supplementary file 1 [file animals-16-02119-s001.zip › animals-4321148-supplementary/animals-4321148-supplementary7.3/Criminal Judgment of Case 32.pdf]

案例 32 刑事判决书

案　　　　由：侵犯公民人身权利、民主权利罪/故意伤害罪
妨害社会管理秩序罪/扰乱公共秩序罪/寻衅滋事罪

案情：

一、故意伤害罪

2017 年 9 月 11 日下午，被告人张某某与张某因琐事发生争吵继而厮打，打斗中，张某某致张某嘴部损伤，其伤情经鉴定构成轻伤二级。

二、寻衅滋事罪

2020 年 1 月 4 日，被告人张某某在其家门口被张某饲养的黑狗扑倒，遂后张某某持铁锹追打黑狗至张某住宅院内，并将张某饲养的其他三只宠物狗打死两只打伤一只。经评估，被打死的两只宠物狗价值 2566 元。

辩护意见：被告人因被被害人家饲养的黑狗扑倒致伤，后在追打黑狗的过程中将被害人家饲养的其他狗打死打伤，其行为不属于无事生非的寻衅滋事行为，应以故意毁损他人财物行为予以评价，因毁损财物价值 2566 元，故其行为不构成犯罪。

判决：被告人张某某故意伤害他人身体致一人轻伤二级，其行为构成故意伤害罪。被告人张某某借故生非，任意毁损公私财物，情节严重，其行为构成寻衅滋事罪。被告人张某某一人犯数罪，应数罪并罚。被告人张某某在被被害人张某家饲养的黑狗扑倒后，借故生非，持铁锹追打黑狗至张某居住院内，又将张某拴养在院内的其他宠物狗打死两只打伤一只，经评估，被打死宠物狗价值 2566 元，属“情节严重”，其行为构成寻衅滋事罪，故被告人的有关辩解及辩护人的有关辩护意见均不能成立，不予采纳。被告人张某某犯故意伤害罪，判处有期徒刑七个月；犯寻衅滋事罪，判处拘役四个月，合并后决定执行有期徒刑七个月，缓刑一年。
