# Supplementary material for: Protecting Companion Animals Under Chinese Criminal Law: Current Practice and Future Paths
Source: Animals (Basel). 2026 Jul 8;16(14):2119. doi: 10.3390/ani16142119 (PMC13405461; doi:10.3390/ani16142119)
Supplement: Supplementary file 1 [file animals-16-02119-s001.zip › animals-4321148-supplementary/animals-4321148-supplementary7.3/Criminal Judgment of Case 33.pdf]

案例 33 刑事判决书

案　　　　由：妨害社会管理秩序罪/扰乱公共秩序罪/寻衅滋事罪

案情：2016 年 12 月 5 日，宠物店店主刘某将一只泰迪犬卖给了另一宠物店的杨某，吴某 1（另案处理）从杨某手上将泰迪犬买走后不久，因泰迪犬拉稀，吴某 1 找到刘某咨询治疗方式后，即自行治疗宠物狗，由于宠物狗死亡。12 月 18 日 16 时许，吴某 1 伙同吴某 2（另案处理）、吴某 3、吴某 4、吴某 5 窜到刘某的宠物店内，由吴某 2 将刘某擒住，吴某 1、吴某 4、吴某 5、吴某 3 相继对刘某进行殴打，因吴某 4 发现店内有摄像头，几人遂逃离现场，吴某 2 在离开时将一只狗笼及一扇玻璃门踢坏，狗笼内有一条拉布拉多犬被踢伤，后经治疗无效死亡。经鉴定，刘某伤情为轻伤二级，刘某店内被损坏的物品及被打死的宠物狗价值为 5600 元。

判决：被告人吴某 3、吴某 5、吴某 4 伙同他人随意殴打被害人致轻伤，任意损毁财物价值 5600 元，情节恶劣，其行为均构成寻衅滋事罪。

一、对于被告人吴某 3，判处有期徒刑三年。

二、对于被告人吴某 5，判处有期徒刑二年。

三、对于被告人吴某 4，判处有期徒刑一年六个月。
