# Supplementary material for: Protecting Companion Animals Under Chinese Criminal Law: Current Practice and Future Paths
Source: Animals (Basel). 2026 Jul 8;16(14):2119. doi: 10.3390/ani16142119 (PMC13405461; doi:10.3390/ani16142119)
Supplement: Supplementary file 1 [file animals-16-02119-s001.zip › animals-4321148-supplementary/animals-4321148-supplementary7.3/Criminal Judgment of Case 4.pdf]

案例 4 刑事判决书

案　　　　由：侵犯财产罪/故意毁坏财物罪

案情：2016 年 4 月 3 日 16 时许，被告人刘某因嫌其邻居陈某在大门口外饲养的宠物狗扰民，乘人不备将“敌敌畏”农药投入陈某用于盛装喂狗饮用水的水桶内，陈某用水桶内饮用水喂狗后致六条宠物狗中毒死亡，经鉴定，被毒死宠物狗的价值为 23500 元。

判决：被告人刘某目无国法，故意毁坏他人财物，数额较大，其行为已构成故意毁坏财物罪；判处有期徒刑八个月，缓刑一年。
