# Supplementary material for: Protecting Companion Animals Under Chinese Criminal Law: Current Practice and Future Paths
Source: Animals (Basel). 2026 Jul 8;16(14):2119. doi: 10.3390/ani16142119 (PMC13405461; doi:10.3390/ani16142119)
Supplement: Supplementary file 1 [file animals-16-02119-s001.zip › animals-4321148-supplementary/animals-4321148-supplementary7.3/Criminal Judgment of Case 5.pdf]

案例 5 刑事判决书

案　　　　由：侵犯财产罪/故意毁坏财物罪

案情：2014 年 1 月 27 日上午，被告人胡某乙提出要用毒药毒狗的方法去偷狗，偷了狗后自己吃，被告人胡某甲、胡某丙、胡某丁均表示同意。当天上午，胡某乙驾驶摩托车搭乘胡某丙、胡某丁驾驶摩托车搭乘胡某甲出发，途中，由胡某乙付款，胡某乙、胡某丙一起到药店购买两粒毒狗的药，并由胡某乙购买排骨，胡某丙将毒狗的药和排骨绑在一起。四被告人携带毒狗的药转了一圈后没有找到作案目标，15 时许，四被告人到达某村，发现被害人刘某喂养的白色下司狗，胡某甲提出来下药毒死该狗，胡某甲将包裹在骨头内的毒药丢到马路上，待狗吃下毒药倒地后，胡某乙把狗捡起来放到胡某丁的摩托车上，胡某丙接了一下手又把狗给了胡某甲，胡某甲提着死狗坐上胡某丁驾驶的摩托车逃走。因逃走时被村民发现，胡某甲将狗丢弃在马路边后和胡某丁驾驶摩托车逃离现场，胡某丙走山路逃跑，胡某乙被村民当场抓获。后胡某乙在村民的要求下打电话叫胡某甲返回协商处理该事，胡某甲独自返回现场，途中遇到前来抓他的村民林某和王某甲，林某和王某甲按住胡某甲的头并拉他的衣领返回现场，走了约十多米后，胡某甲捡起一把砌刀反抗，将林某、王某甲砍伤后逃跑。经鉴定：被毒死的狗价值为 8000 元。经鉴定：林某、王某甲的损伤构成轻微伤。

判决：被告人胡某甲、胡某乙、胡某丙、胡某丁故意毁坏他人财物，数额较大，其行为已构成故意毁坏财物罪。

一、对于被告人胡某甲，判处有期徒刑九个月。

二、对于被告人胡某乙，判处有期徒刑九个月。

三、对于被告人胡某丙，判处有期徒刑六个月，缓刑一年。

四、对于被告人胡某丁，判处有期徒刑六个月，缓刑一年。
