# Supplementary material for: Protecting Companion Animals Under Chinese Criminal Law: Current Practice and Future Paths
Source: Animals (Basel). 2026 Jul 8;16(14):2119. doi: 10.3390/ani16142119 (PMC13405461; doi:10.3390/ani16142119)
Supplement: Supplementary file 1 [file animals-16-02119-s001.zip › animals-4321148-supplementary/animals-4321148-supplementary7.3/Criminal Judgment of Case 6.pdf]

案例 6 刑事裁定书

案　　　　由：侵犯财产罪/故意毁坏财物罪

案情：2018 年 1 月 20 日 7 时许，被告人刘某为了盗窃一只中华田园犬，在某门口扔了含有毒物质的鸭脖子给其食用，先后导致被害人余某饲养的一只中华田园犬和一只罗威纳犬食用后当场死亡。经鉴定，死亡的中华田园犬和罗威纳犬分别价值人民币 800 元、11000 元。

一审判决：被告人刘某为盗窃他人狗只而采用毒死他人狗只的方法致他人财物毁坏价值人民币 11800 元，数额较大，其行为已构成故意毁坏财物罪；判处有期徒刑一年。

二审裁定：上诉人刘某为实施盗窃而采用投掷毒物的方法致他人饲养的宠物犬中毒死亡，致他人财物损失价值人民币 11800 元，数额较大，其行为已构成故意毁坏财物罪。原审判决认定事实清楚，证据确实、充分，定罪准确，量刑适当，审判程序合法。上诉人刘某请求改判的上诉理由不能成立，不予采纳。驳回上诉，维持原判。
