# Supplementary material for: Protecting Companion Animals Under Chinese Criminal Law: Current Practice and Future Paths
Source: Animals (Basel). 2026 Jul 8;16(14):2119. doi: 10.3390/ani16142119 (PMC13405461; doi:10.3390/ani16142119)
Supplement: Supplementary file 1 [file animals-16-02119-s001.zip › animals-4321148-supplementary/animals-4321148-supplementary7.3/Criminal Judgment of Case 7.pdf]

案例 7 刑事判决书

案　　　　由：侵犯财产罪/盗窃罪
侵犯财产罪/故意毁坏财物罪

案情：

一、盗窃罪

2014 年 11 月，被告人孙某、程某、汪某、徐某甲商议盗窃农户家的狗，由孙某在网上购买弓弩和毒镖，用汪某的驾驶证租赁了一辆轿车，并由孙某与被告人张某商议，由张某收购盗得的狗。被告人孙某、程某、汪某、徐某甲驾车，利用弓弩发射毒镖的方式将狗射死，后盗走，并将盗得的七条狗卖给被告人张某，总价值 3075 元。

二、故意毁坏财物罪

1.2014 年 11 月下旬的一天下午，被告人孙某、程某、汪某、徐某甲驾车来到某地，利用弓弩发射毒镖的方式射死丁某某家一条狗。因被人发现，该狗未盗走。经鉴定，该狗价值 525 元。

2.2014 年 12 月 7 日下午，被告人孙某、程某、汪某、徐某甲驾车来到某地，利用弓弩发射毒镖的方式射死张某乙家一条秋田犬。因院内人多，该狗未盗走。经鉴定，该狗价值 10000 元。

判决：被告人孙某、程某、汪某、徐某甲以非法占有为目的，盗窃公私财物，数额较大，四被告人行为均已构成盗窃罪。被告人张某、张某甲事前与孙某通谋，事后对盗窃所得的赃物予以收购，是盗窃罪共犯，二被告人行为亦构成盗窃罪。被告人孙某、程某、汪某、徐某甲为了实现其盗窃他人财物的目的，采取破坏性手段，其行为牵连触犯了故意毁坏财物罪和盗窃罪，依法应择一重罪处罚。被告人故意毁坏财物，数额较大，且系既遂，罪行较重，而盗窃，系未遂，罪行较轻，故应以故意毁坏财物罪追究四被告人的刑事责任。公诉机关指控罪名成立。被告人孙某、程某、汪某、徐某甲身犯二罪，依法应实行数罪并罚。

一、被告人孙某犯盗窃罪，判处有期徒刑六个月，并处罚金 4000 元；犯故意毁坏财物罪，判处有期徒刑六个月，决定合并执行有期徒刑九个月，缓刑一年六个月，并处罚金 4000 元；

二、被告人程某犯盗窃罪，判处有期徒刑六个月，并处罚金 4000 元；犯故意毁坏财物罪，判处有期徒刑六个月，决定合并执行有期徒刑九个月，并处罚金 4000 元；

三、被告人汪某犯盗窃罪，判处有期徒刑六个月，并处罚金 4000 元；犯故意毁坏财物罪，判处有期徒刑六个月，决定合并执行有期徒刑九个月，缓刑一年，并处罚金 4000 元；

四、被告人徐某甲犯盗窃罪，判处有期徒刑六个月，并处罚金 4000 元；犯故意毁坏财物罪，判处有期徒刑六个月，决定合并执行有期徒刑九个月，缓刑一年，并处罚金 4000 元；

五、被告人张某犯盗窃罪，判处拘役三个月，缓刑十个月，并处罚金 3000 元；

六、被告人张某甲犯盗窃罪，判处拘役二个月，缓刑八个月，并处罚金 2000 元；

七、作案工具弓弩一只，予以没收。
