# Supplementary material for: Protecting Companion Animals Under Chinese Criminal Law: Current Practice and Future Paths
Source: Animals (Basel). 2026 Jul 8;16(14):2119. doi: 10.3390/ani16142119 (PMC13405461; doi:10.3390/ani16142119)
Supplement: Supplementary file 1 [file animals-16-02119-s001.zip › animals-4321148-supplementary/animals-4321148-supplementary7.3/Criminal Judgment of Case 8.pdf]

案例 8 刑事判决书

案　　　　由：侵犯财产罪/故意毁坏财物罪

案情：被告人涂某称因其想吃狗肉，便萌生了偷狗的想法。2019 年 7 月 21 日凌晨 0 时多，涂某驾驶摩托车窜至被害人何某家门口，发现门口有一只体型较大的狗（罗威纳），便拿出事先准备好的毒镖针装在弓弩上朝狗身上射去，后离开现场。涂某再次回到现场时发现该狗已经死亡，当其准备将狗盗走时被何某增发现并追赶，涂某逃离现场。

判决：被告人涂某故意毁坏他人财物，数额较大，其行为已构成故意毁坏财物罪；判处有期徒刑十个月。
