# Supplementary material for: Protecting Companion Animals Under Chinese Criminal Law: Current Practice and Future Paths
Source: Animals (Basel). 2026 Jul 8;16(14):2119. doi: 10.3390/ani16142119 (PMC13405461; doi:10.3390/ani16142119)
Supplement: Supplementary file 1 [file animals-16-02119-s001.zip › animals-4321148-supplementary/animals-4321148-supplementary7.3/Criminal Judgment of Case 9.pdf]

案例 9 刑事判决书

案　　　　由：侵犯财产罪/故意毁坏财物罪

案情：2017 年 2 月 23 日 5 时 17 分，被告人赵某、于某夫妇驾驶轿车时，见某店主李某的两只柯基犬（价值 24100 元）在门前，遂产生盗窃之念。赵将事先准备好的绑有毒药的食物投向柯基犬后，二人驾车继续前行一段距离。于先行下车步行返回欲捡拾柯基犬时，因两只柯基犬进入宠物店而未能得逞。赵又掉头驾车返回，亦未捡拾到柯基犬后二人驾车驶离现场。因赵、于二人投放带有毒药的食物，导致两只柯基犬进食后中毒死亡。经鉴定：两只犬心血及胃内容物均检出氰化物成分，起获的白色柱状物毒药中检出氰离子成分。

判决：被告人赵某、于某采取投放带有氰化物成分的食物为手段，欲盗窃他人柯基犬，因意志以外的原因未能得逞，并因而造成价值 24100 元的柯基犬死亡，数额较大，其行为已构成故意毁坏财物罪。

一、对于被告人赵某，判处有期徒刑二年，缓刑三年。

二、对于被告人于某，判处有期徒刑二年，缓刑三年。
